# Immunoglobulin A Subtype and Survival After Upfront Autologous Stem Cell Transplantation in Multiple Myeloma: A Real-World Cohort from the Bortezomib Era

**DOI:** 10.3390/cancers18193178

**Published:** 2026-10-02

**Authors:** Tuba Güllü Koca, Nizameddin Koca, Fazıl Çağrı Hunutlu, Ahmet Mert Yanık, Sinem Çubukçu, Rümeysa Yılmaz, Tuba Ersal, Vildan Gürsoy, İbrahim Ethem Pınar, Vildan Özkocaman, Fahir Özkalemkaş

**Affiliations:** 1 Department of Hematology, Bursa Şehir Training & Research Hospital, University of Health Sciences, 16250 Bursa, Türkiye; tubagullukoca@uludag.edu.tr (T.G.K.); fazilcagri@uludag.edu.tr (F.Ç.H.); ahmet.mert@marmara.edu.tr (A.M.Y.); 2 Department of Internal Medicine, Bursa Şehir Training & Research Hospital, University of Health Sciences, 16250 Bursa, Türkiye; 3 Department of Hematology, Ali Osman Sönmez Oncology Hospital, 16020 Bursa, Türkiye; sinemcubukcu@uludag.edu.tr; 4 Department of Hematology, Bursa Uludağ University Hospital, Bursa Uludağ University, 16059 Bursa, Türkiye; rumeysayilmaz@uludag.edu.tr (R.Y.); tubaersal@uludag.edu.tr (T.E.); vildangursoy@uludag.edu.tr (V.G.); iethempinar@uludag.edu.tr (İ.E.P.); vildanoz@uludag.edu.tr (V.Ö.); fahir@uludag.edu.tr (F.Ö.)

**Keywords:** multiple myeloma, immunoglobulin A, autologous stem cell transplantation, prognosis, cytogenetics, risk stratification

## Abstract

Background: Immunoglobulin isotype is recorded at diagnosis in every patient with intact-immunoglobulin multiple myeloma, but is not retained in the ISS, R-ISS, or R2-ISS. Methods: We analyzed 196 consecutive patients with IgA or IgG myeloma who underwent upfront autologous stem cell transplantation at a single center (2009–2023). The primary endpoint was progression-free survival (PFS); overall survival (OS) was the secondary endpoint. Cox models used information available at transplantation, including the induction regimen and transplant year, in the complete-case set (*n* = 131) and after multiple imputation (*n* = 196). Results: Median PFS was 14.8 versus 30.5 months and OS 43.2 versus 94.2 months in IgA versus IgG patients (both *p* < 0.001). IgA isotype was associated with inferior PFS (adjusted HR 1.85, 95% CI 1.13–3.02; imputed HR 1.83, 95% CI 1.22–2.73). For OS, the HR was 1.68 (0.98–2.88) in the complete-case analysis and 1.91 (1.24–2.94) after imputation, so its magnitude is uncertain. Models with maintenance gave 1.87 and 1.82. Progression documentation differed by maintenance status. Cytogenetic data were too incomplete to assess independence from genomic risk. Conclusions: In a bortezomib-era transplant cohort, IgA isotype was associated with inferior PFS; the association with OS was of uncertain magnitude. The finding is hypothesis-generating and requires validation in cohorts with complete cytogenetic characterization.

## Simple Summary

Multiple myeloma is a cancer of plasma cells, which secrete an abnormal antibody. The class of that antibody is identified in every patient during routine diagnostic work-up at no additional cost, but it plays no part in the scores used to assess disease aggressiveness. We reviewed 196 patients who received an autologous stem cell transplant at a single center between 2009 and 2023, all of whom were treated with bortezomib-based induction before anti-CD38 antibodies became routine first-line use. Patients whose myeloma produced immunoglobulin A progressed and died sooner than those producing immunoglobulin G. After accounting for stage, age, depth of response before transplantation, induction treatment, and year of transplantation, the difference in progression remained, while the difference in survival was smaller and less certain. Chromosomal testing was too incomplete in this cohort to determine whether the difference reflects known genetic risk factors, and progression was recorded differently between patient groups, so overall survival is the more dependable of our two endpoints. We do not propose that the antibody class should change treatment. We suggest that it be recorded and tested in larger studies with complete genetic characterization, where its value, if any, can be established.

## 1. Introduction

An estimated 35,780 new cases of multiple myeloma were projected in the United States in 2024 [1], and the disease accounts for approximately 10% of hematologic malignancies [2]. Over the past two decades, treatment has changed substantially with successive generations of proteasome inhibitors, immunomodulatory agents, anti-CD38 monoclonal antibodies, and, most recently, chimeric antigen receptor T-cell products and bispecific antibodies [2]. In transplant-eligible patients, upfront autologous stem cell transplantation (ASCT) following bortezomib-based induction remains a cornerstone of therapy [2,3], and lenalidomide maintenance after ASCT is standard of care, based on randomized trials and a subsequent individual-patient data meta-analysis [4,5,6].

Risk stratification is based on the International Staging System (ISS) and its revisions [7,8,9]. The Revised ISS incorporates del(17p), t(4;14), and t(14;16), along with serum lactate dehydrogenase (LDH), thereby identifying a high-risk subgroup with a 5-year overall survival of 40% [8]. Immunoglobulin subtype was recorded in the cohorts from which these models were derived, but was not retained as a component of the final scores. Whether that omission costs discriminatory power specifically in the post-transplant setting has not been systematically evaluated.

IgA myeloma constitutes approximately 20–25% of newly diagnosed cases [2]. Several considerations suggest its behavior may differ from that of IgG disease. The IgA isotype is associated with t(4;14) [10], a translocation arising from class-switch recombination errors at the immunoglobulin heavy chain locus, which raises the possibility of isotype-specific bias in the translocation repertoire more broadly. Immunoparesis is an established adverse prognostic factor in myeloma, and recovery of uninvolved immunoglobulins after transplantation is associated with better outcomes [11]; whether immunoparesis is deeper or more persistent in IgA than in IgG disease has not been established. Separately, IgA monoclonal proteins co-migrate with the beta fraction on serum protein electrophoresis and are quantified with unreliable precision, so that response and progression may be measured differently in IgA than in IgG disease [12]. Most ASCT outcome series are not powered for subtype-specific analysis, and existing data are largely confined to subgroup tables rather than primary analyses [13].

We therefore analyzed IgA versus IgG myeloma outcomes in a real-world cohort of 196 patients who underwent upfront ASCT between 2009 and 2023. Our primary objective was to determine whether the IgA subtype is associated with inferior progression-free and overall survival after adjustment for established prognostic factors. We further examined whether any such disadvantage was observable in patients without detectable t(4;14) and whether it has narrowed in the lenalidomide maintenance era.

Every patient in this cohort was treated with a proteasome inhibitor backbone before anti-CD38 antibodies entered routine first-line use. This defines the study’s setting in two ways. It limits how far the absolute survival estimates can be carried into contemporary quadruplet-treated practice. It also reduces a source of confounding: in cohorts spanning the current transition, quadruplet, triplet, and doublet induction coexist, access varies between and within health systems, and treatment intensity is strongly correlated with calendar time and center, so an isotype effect can only be estimated after adjustment for a covariate that is itself a proxy for era and access. A cohort treated on a common backbone narrows that problem, although, as we show, it does not remove treatment heterogeneity altogether. It also resembles current practice in many health systems where anti-CD38 antibodies are not reimbursed as first-line therapy.

## 2. Materials and Methods

### 2.1. Study Design and Population

We conducted a retrospective cohort study of consecutive patients with IgA or IgG multiple myeloma who underwent upfront ASCT at Bursa Uludağ University Hospital between January 2009 and October 2023 (the transplant accrual period; the latest follow-up date recorded in the registry was 29 April 2026, Section 2.2). The institutional transplant registry contained 337 records. We excluded 60 records with light-chain-only myeloma, 47 in which the isotype was not recorded, 6 with IgM or other rare subtypes, and 1 with missing survival follow-up, leaving 223 transplant records.

The unit of analysis was the patient, indexed at the first (upfront) transplant. Because the registry records transplant episodes rather than patients, we excluded 20 duplicate entries for the same transplant and 7 records of a second transplant in patients already included; all 7 s transplants were salvage procedures after documented progression. Planned tandem transplantation was not an institutional practice during the study period, and post-transplant consolidation was not given. The analytical cohort comprised 196 patients: 47 with IgA myeloma (24.0%) and 149 with IgG myeloma (76.0%) (Figure S1).

Upfront ASCT was defined as a first autologous transplant given as consolidation of the initial bortezomib-based induction, without a documented progression before transplantation. The number of induction cycles and any change of regimen before transplantation were not recorded as discrete fields, so the number of prior lines cannot be reported. In one patient, the recorded relapse date preceded the transplant and coincided with the month of diagnosis; it could not be verified against source documents and was handled by the rule for unusable progression dates set out in Section 2.2. The interval from diagnosis to transplantation exceeded 24 months in some patients, more often in the IgA group (Section 3.1). The main reason was not clinical: during several periods within the study years, high-dose melphalan could not be obtained in Türkiye or its supply was delayed, and transplants were postponed until the drug became available. Such delays were unrelated to isotype and are an additional reason why calendar time carries information in this cohort (Section 3.7). Eligibility for ASCT followed institutional guidelines consistent with international recommendations: generally age 70 years or younger, ECOG performance status 2 or better, and absence of organ dysfunction precluding high-dose melphalan [3].

The excluded groups are characterized in Table S4. The 60 light-chain-only records corresponded to 58 patients transplanted over the entire study period (median 2017), with outcomes similar to those of the analytical cohort. Of the 47 records without a recorded isotype, 24 had no transplant date and represent incomplete registry entries rather than transplanted patients; the remaining 23 records corresponded to 22 patients, transplanted mainly from 2017 onwards (median 2021.5), in whom ISS stage was recorded in only 7. These were therefore incompletely entered records rather than a clinically distinct group; the reason the isotype field was left blank is not documented, and we treat these as missing data at the registry level rather than as an exclusion on clinical grounds.

The study is reported in accordance with the Strengthening the Reporting of Observational Studies in Epidemiology (STROBE) statement for cohort studies [14]; the completed checklist is provided as a supplementary file.

### 2.2. Endpoints and Definitions

The primary endpoint was PFS, defined as the time from ASCT to first documented progression or death from any cause, whichever occurred first. The event algorithm was as follows: a patient with a documented progression contributed a PFS event at the progression date; a patient who died without a documented progression contributed a PFS event at the date of death; a patient alive without a documented progression was censored at the date of the last hospital visit recorded in the registry. This field contains the most recent hospital contact and does not specify whether disease status was evaluated at that visit; therefore, it serves as an operational censoring proxy, and we cannot verify that an adequate progression assessment was made on the censoring date for every patient. Deaths were never censored. The consequence of an unrecorded progression is therefore a later event date (the date of death) or, in a living patient, censoring, and in both cases, measured PFS is lengthened, not shortened (Section 3.6).

A recorded progression date was used only if it fell on or after the date of transplantation and on or before the date of death or the latest date recorded in the registry. A progression recorded on the same date as death was counted as a documented progression. Three recorded progression dates fell outside this window: one before transplantation (the patient noted in Section 2.1) and two after the recorded date of death. None could be corrected from the source documents. These dates were not moved or imputed; the three patients, all of whom died, were classified as deaths without documented progression, with the PFS event at the date of death. None of the three was among the complete cases used in the multivariable models, and their exclusion is examined in Section 3.7. Relapse and maintenance status were both missing for one further patient, who died and is counted as a death without documented progression. The first-event classification, the comparisons of progression documented before death, the survival models, and the post-progression analysis were all generated from this single finalized dataset (Section 3.6).

Progression was determined by treating physicians according to the International Myeloma Working Group consensus criteria [15], applied contemporaneously; no retrospective re-adjudication was performed. Response was assessed with the standard myeloma laboratory panel (serum protein electrophoresis with immunofixation, quantitative immunoglobulins, serum free light chains, and urine studies, with bone marrow examination where required to confirm complete response) together with PET-CT. The institutional schedule called for assessment at the end of induction, on day +100 after transplantation, and every 3 months thereafter; the same panel was used to identify progression, which could therefore be biochemical or imaging-defined. This was the intended schedule. The registry recorded the dates of transplantation, progression, death, and last hospital visit, but not the dates or results of individual assessments, so adherence to the schedule could not be verified, and the actual intensity of surveillance for individual patients is unknown. Assessments were not retrospectively harmonized across the study period, and the registry did not record the modality by which each progression was detected. Overall survival (OS) was defined as the time from ASCT to death from any cause.

Vital status and the date of death were taken from hospital records and from the national civil registration system (MERNİS), which records all deaths in Türkiye. Transplant accrual ended in October 2023 (the last transplant in the cohort was performed in May 2023), and the latest date recorded in the registry was 29 April 2026. No dated record of a cohort-wide vital-status query was retained, so survival through a common date cannot be verified for every living patient. Patients alive were therefore censored for OS at the last date they were documented to be alive: the last recorded hospital visit or, in one patient whose progression was recorded later than the last-visit date, the progression date. For 68 of the 86 living patients, this date was within 6 months of 29 April 2026; for 15, it was more than 12 months earlier (at most 179 months earlier), and these patients were lost to follow-up after that date. An analysis in which all living patients were instead assumed to be alive on 29 April 2026, or on later dates, is reported as a sensitivity analysis (Section 2.5). Two measures of follow-up are reported: the observed time from transplantation to death or to the last documented date of survival (Section 3.1), and the potential follow-up estimated using the reverse Kaplan–Meier method [16] (Section 3.7). Pre-ASCT response depth was coded ordinally (complete response [CR] = 1, very good partial response [VGPR] = 2, partial response [PR] = 3), with higher values indicating a shallower response; categorical coding was examined in sensitivity analyses. The treatment era was a binary variable split at 2015, when lenalidomide maintenance became routine at our center.

### 2.3. Treatment Protocol

All patients received bortezomib-based induction. The regimen was determined by drug availability and reimbursement at the time of treatment rather than by a written institutional protocol, and the intensity of induction varied accordingly across the study period. Among the 195 patients with a recorded induction regimen, 103 (52.8%) received bortezomib and dexamethasone, 25 (12.8%) received bortezomib, cyclophosphamide, and dexamethasone (VCD), and 67 (34.4%) received bortezomib, lenalidomide, and dexamethasone (VRD) or another immunomodulatory-drug-containing triplet. Among transplanted patients, VRD-type triplets first appeared from 2012 onwards and VCD from 2015 onwards, whereas bortezomib and dexamethasone were in use throughout the study period and predominated until the mid-2010s. One patient (0.5%), transplanted in 2023, received a daratumumab-containing regimen before transplantation; the cohort therefore almost entirely predates routine anti-CD38 antibody use. Induction regimen did not differ between isotypes (*p* = 0.665; Section 3.1).

Patients were referred for transplantation if they achieved at least a partial response to induction and had no contraindication to high-dose therapy. A VGPR or better was preferred but not required, and 17 of the 146 patients with an evaluable pre-transplant assessment (11.6%) proceeded to transplantation in PR. Conditioning was melphalan 200 mg/m^2^, reduced to 140 mg/m^2^ in patients with renal impairment or significant comorbidity; 9 of 194 patients with a recorded dose (4.6%) received the reduced dose.

Lenalidomide maintenance was recorded in the registry as ever received (yes or no); the date of the first maintenance dose was not recorded. Institutional practice from 2015 onwards was to start maintenance after hematological recovery, about 3 months after transplantation, and to continue until progression or intolerance. Because the variable describes eventual receipt rather than treatment status at a defined time point, it is a post-baseline exposure; this is handled in Section 2.5.

### 2.4. Cytogenetic Analysis

Cytogenetic risk was assessed by interphase fluorescence in situ hybridization (iFISH) on bone marrow aspirates obtained at diagnosis, with probes for t(4;14), t(14;16), and del(17p). Hybridization was performed on an unselected bone marrow aspirate; CD138 immunomagnetic enrichment of plasma cells was not part of the routine laboratory workflow during the study period. Two hundred interphase nuclei were scored per probe, and a result was called positive when the abnormality was present in at least 10% of scored nuclei, a threshold that, applied to unselected marrow, favors specificity over sensitivity. The panel did not include 1q21 gain or amplification, del(1p32), biallelic TP53 inactivation, or conventional karyotyping.

The registry recorded each probe as present, absent, or not performed, and the field could also be left blank. A probe was counted as tested only when a result (present or absent) was recorded; probes coded as not performed and blank fields were both treated as untested, never as negative. The distribution of the four categories for each probe-isotype pair is given in Table S8. Testing was incomplete and uneven across probes: t(4;14) was assessed in 64 patients (32.7%; 17 IgA, 47 IgG), whereas del(17p) and t(14;16) were assessed in only 21 (10.7%) and 20 (10.2%) patients, respectively, and all three probes in 20 (10.2%). Testing became more frequent over time and differed by probe: the t(4;14) probe was in use from 2012 (14.5% of patients transplanted before 2015 and 41.0% thereafter were tested), whereas del(17p) and t(14;16) were first assessed in patients transplanted in 2015 and remained infrequent even after that (21 and 20 patients in total). Cytogenetic analyses are therefore confined to t(4;14), are exploratory, and cannot address whether the isotype association is independent of genomic risk as defined by current consensus [17]. To assess whether the tested subgroup was representative, PFS was compared between tested and untested patients within each isotype by the log-rank test, and a testing-by-subtype interaction was fitted.

### 2.5. Statistical Analysis

Baseline characteristics were compared using the Wilcoxon rank-sum test, chi-square or Fisher’s exact test, and the balance between isotypes was additionally described using standardized mean differences, with values below 0.2 conventionally considered small [18]. Survival was estimated using the Kaplan–Meier method and compared with the log-rank test; 95% confidence intervals and the number at risk are provided for all point estimates. Multivariable Cox proportional hazards regression was performed in the complete-case analysis. The pre-specified model (the base model) included IgA subtype, ISS stage (continuous), age at transplantation, pre-ASCT response depth (ordinal), and lenalidomide maintenance use. In a pre-specified sensitivity analysis, the model was extended to include serum LDH measured before conditioning (log2-transformed) and melphalan dose. Univariable hazard ratios were computed for all evaluated clinical factors on the full cohort, with the evaluable denominator and the number of events reported for each (Section 3.2).

Because maintenance is received after the time origin and its start date was not recorded, a covariate for eventual receipt can introduce immortal-time bias [19]. We therefore report, alongside the base model, a model restricted to information available at transplantation (IgA subtype, ISS stage, age, and pre-ASCT response), and the maintenance covariate in the base model is interpreted as a marker of follow-up intensity rather than as a treatment effect (Section 3.6), recognizing that this interpretation does not remove the temporal problem. During peer review, we added a baseline-only model that combines the information available at transplantation—isotype, ISS stage, age, response depth, induction regimen, and calendar year of transplantation—without maintenance, fitted in complete cases and via multiple imputation (see below). This model was not pre-specified. Because it avoids the post-baseline covariate and accounts for the treatment period, it is the primary basis for our interpretation of the post-transplant association (Section 3.2); the pre-specified base model and the other models containing maintenance are reported as secondary analyses (Table 2B and Table S3). Further sensitivity models, all fitted in the same 131 complete cases unless stated, coded ISS stage and response depth as categorical variables, added induction regimen and calendar year of transplantation, added light-chain type, and omitted response depth so that a larger sample (*n* = 179) could be used; the last was also refitted in the 131 complete cases to separate the effect of the covariate from that of sample restriction (Table S3). Two further sensitivity analyses addressed the event data: exclusion of the three patients with an unusable progression date (Section 2.2), and censoring of living patients at a common date (29 April 2026, and 1 to 12 months later) instead of at the last documented date alive (Table S9). The latter analysis assumes that every living patient survived to the chosen date. It holds all deaths and death dates fixed and lengthens only the censored follow-up of living patients; it is not an additional observed follow-up.

Of 196 patients, 131 had complete data for all base-model covariates (the same 131 patients were complete for the baseline-only model). Missing data were dominated by the pre-transplant response assessment, absent for 50 patients (25.5%), most of whom were transplanted before structured response documentation became routine; ISS stage was missing for 15, and age and maintenance status for 1 each (Table S1); of the 65 excluded patients, 50 lacked the response assessment and 15 had a recorded response but lacked ISS stage (14) or age (1). Complete-case status was not associated with isotype (36.2% of IgA and 32.2% of IgG patients excluded; *p* = 0.723), but excluded patients were transplanted earlier and more often received a doublet induction; a full comparison of included and excluded patients is given in Table S6. Complete-case analysis assumes that missingness is unrelated to outcome given the covariates, an assumption that cannot be verified. As a sensitivity analysis we therefore performed multiple imputation by chained equations (20 imputed datasets) [20,21], with an imputation model containing isotype, sex, age, ISS stage, response depth, maintenance, treatment era, induction regimen, LDH, melphalan dose, light-chain type, and the event indicator and Nelson–Aalen cumulative hazard for each outcome [22] (for the baseline-only model, calendar year of transplantation in place of treatment era); estimates were combined by Rubin’s rules. Imputation does not remove bias from missingness that depends on unobserved values, and we present it as a check on the complete-case estimate rather than as a correction of it.

The proportional hazards assumption was assessed by Schoenfeld residuals, globally and for each covariate, in the PFS and OS models separately (Table S5). Where it was violated, a stratified model was fitted as a sensitivity analysis, and a model with a time-varying coefficient for age was also examined in which the age term was supplemented by an age × log(t + 1) interaction, with t in months since transplantation (a time-transform term in the survival package); this model was fitted for PFS and OS, both in the base model and in the baseline-only model. The incremental discrimination of the isotype term was quantified using Harrell’s concordance index and compared between nested models fitted to the same patients, with the likelihood ratio test as the formal comparison. Because apparent discrimination is optimistic when measured on the data used to fit the model, the concordance index was internally validated using a bootstrap optimism correction (200 resamples) [23]. A bootstrap percentile interval was obtained for the apparent difference between the models with and without isotype; the optimism-corrected difference is reported as a point estimate. No external validation was available. To quantify how strong unmeasured confounding, for example, by cytogenetic lesions that were not assessed, would have to be to explain the observed association, E-values were computed for the adjusted hazard ratios and for the confidence limits closest to the null, after converting hazard ratios to approximate risk ratios because the outcomes are common [24]. Pre-specified interaction tests (IgA by maintenance; IgA by ISS stage) evaluated effect modification; subgroup and interaction analyses are exploratory.

Two landmark analyses were performed to address time-related bias in maintenance assignment [25]. In each, patients who were alive and progression-free at the landmark time were retained; survival time was reset to zero at the landmark; and events occurring before the landmark were excluded from both the numerator and the denominator; patients progressing or dying before the landmark contribute to neither group. Because the maintenance start date was not recorded, the landmark analyses classify patients by eventual receipt rather than by documented treatment status at the landmark; they exclude early events but do not fully resolve the time dependence of the exposure. Kaplan–Meier estimates and log-rank tests from each landmark use all eligible patients; the adjusted Cox models use the complete cases among them. Both a 6-month and a 12-month landmark were examined, the second to assess sensitivity to the choice of landmark time. Era-stratified analyses (pre-2015 versus 2015 onwards) examined the effect of the adoption of lenalidomide maintenance. All analyses were performed in R version 4.6 [26] with the packages survival [27], survminer, and mice [20]. Two-sided *p*-values below 0.05 were considered significant for pre-specified analyses; exploratory analyses are reported with uncorrected *p*-values, and *p*-values are given to three decimal places or as *p* < 0.001.

### 2.6. Use of Generative Artificial Intelligence

Generative artificial intelligence tools were used for language editing (Grammarly; Claude, Anthropic) and for assistance with the analysis code (Claude Opus 5.5, Anthropic, San Francisco, CA, USA). The authors designed the study, made all analytical decisions, verified all results, and take full responsibility for the content of the manuscript, the integrity of the data, and the conclusions drawn.

## 3. Results

### 3.1. Patient Characteristics

The analytical cohort comprised 196 patients: 47 with IgA myeloma (24.0%) and 149 with IgG myeloma (76.0%). Baseline characteristics are shown in Table 1. Median age at transplantation was 58.6 years (IQR 52.9–63.7), and 91 patients (46.4%) were female. ISS stage III disease was present in 98 of 181 evaluable patients (54.1%), and 81 of 146 patients with an evaluable pre-transplant assessment (55.5%) were in complete response at the time of transplantation. Lenalidomide maintenance was given to 132 of 195 patients (67.7%), and 134 transplants (68.4%) were performed from 2015 onwards.

The two groups were similar in distribution for most characteristics. Age, sex, ISS stage, time from diagnosis to transplantation, induction regimen, melphalan dose, creatinine, CD34+ cell dose, neutrophil engraftment, era, and the rate of t(4;14) testing did not differ significantly, and their standardized mean differences did not exceed 0.24. Three variables differed. Lambda light chains were more frequent in IgA than in IgG disease (48.9% versus 29.5%; *p* = 0.021; standardized difference 0.41). Pre-transplant response was deeper in IgA patients: complete response in 24 of 34 evaluable IgA patients (70.6%) versus 57 of 112 IgG patients (50.9%); the omnibus test across CR, VGPR, and PR gave *p* = 0.030, and the comparison of CR against less than CR gave *p* = 0.050. Serum LDH before conditioning was modestly higher in IgA patients (median 212 versus 185 U/L; *p* = 0.027). Maintenance was numerically more frequent in IgA patients (78.3% versus 64.4%; *p* = 0.104; standardized difference 0.31). The difference in response is notable because it favors IgA patients, who nonetheless fared substantially worse.

### 3.2. IgA Subtype and Survival

**Figure 1:**
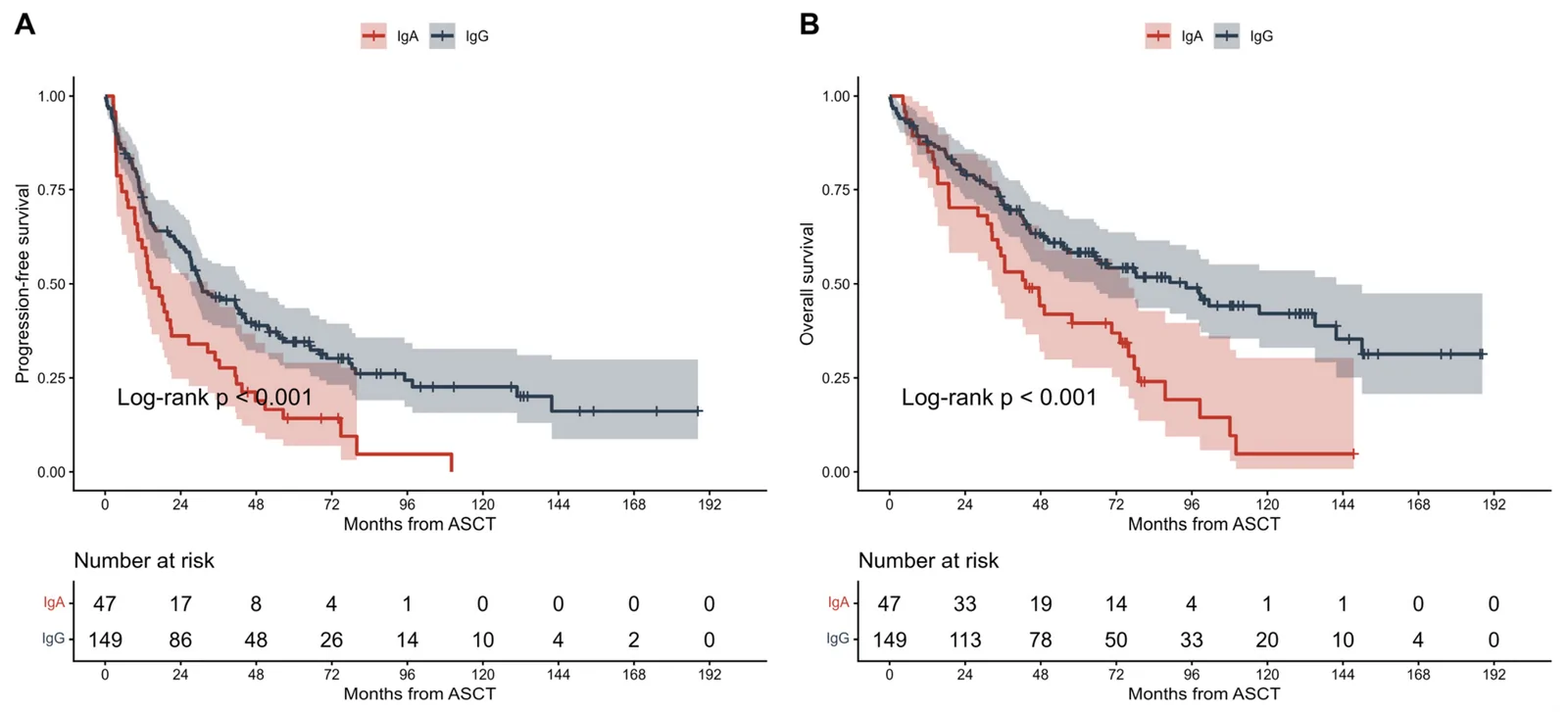
Kaplan–Meier estimates of (**A**) progression-free and (**B**) overall survival by myeloma subtype in the full cohort (*n* = 196). IgA patients (*n* = 47) had shorter PFS (median 14.8 versus 30.5 months; *p* < 0.001) and OS (median 43.2 versus 94.2 months; *p* < 0.001). Shaded areas denote 95% confidence intervals; numbers at risk are shown below each panel. Patients alive were censored at the last recorded hospital visit for PFS and at the last documented date alive for OS.

Median PFS was substantially shorter in IgA patients: 14.8 months (95% CI 10.4–32.6) versus 30.5 months (95% CI 26.8–44.5) for IgG (log-rank *p* < 0.001; Figure 1A), with 43 PFS events among 47 IgA patients and 104 among 149 IgG patients. The 2-year PFS rate was 36.2% (95% CI, 24.7–52.9) versus 59.9% (95% CI, 52.4–68.3), and the 5-year rate was 14.2% (95% CI, 6.9–29.1) versus 34.6% (95% CI, 27.5–43.6). Overall survival followed the same pattern: median 43.2 months (95% CI 32.6–75.9) versus 94.2 months (95% CI 64.9–141.9) (*p* < 0.001; Figure 1B), with 37 and 73 deaths respectively, and 5-year OS of 39.6% (27.7–56.7) versus 58.3% (50.6–67.2).

The main multivariable analysis was the baseline-only model, adjusted for ISS stage, age, pre-ASCT response depth, induction regimen, and calendar year of transplantation (Table 2A; *n* = 131 complete cases, 30 IgA and 101 IgG; 95 PFS events, 27 in IgA patients; 69 deaths, 22 in IgA patients). IgA subtype was associated with inferior PFS (HR 1.85, 95% CI 1.13–3.02; *p* = 0.015); for OS, the hazard ratio was 1.68 (95% CI 0.98–2.88; *p* = 0.058). Using multiple imputation for all 196 patients (147 PFS events, 110 deaths), the estimates were 1.83 (95% CI, 1.22–2.73; *p* = 0.004) for PFS and 1.91 (95% CI, 1.24–2.94; *p* = 0.004) for OS. The complete-case and imputed OS estimates differ in whether their confidence intervals include unity; we read this as uncertainty that reflects the information available and the assumptions made about missing data, and neither analysis resolves it on its own.

In the secondary analyses (Table 2B), the pre-specified base model, which adjusted for ISS stage, age, pre-ASCT response depth, and eventual receipt of lenalidomide maintenance, gave hazard ratios of 1.87 (95% CI 1.17–3.01; *p* = 0.010) for PFS and 1.82 (95% CI 1.07–3.09; *p* = 0.026) for OS. Without the maintenance covariate and without adjustment for treatment period, the estimates were 2.06 (95% CI 1.29–3.30; *p* = 0.003) and 1.79 (95% CI 1.07–3.00; *p* = 0.026) in the same 131 patients.

Adding the isotype term to the model containing ISS stage, age, response depth, and maintenance improved the fit according to the likelihood ratio test (PFS *p* = 0.013, ΔAIC 4.2; OS *p* = 0.031), but the gain in discrimination was small. The apparent concordance index rose from 0.604 to 0.619 for PFS and from 0.603 to 0.622 for OS; after bootstrap optimism correction, the values were 0.584 and 0.596 for PFS and 0.574 and 0.591 for OS, an optimism-corrected difference of 0.012 and 0.017. The apparent differences were 0.014 for PFS (95% bootstrap interval: −0.011 to 0.052) and 0.019 for OS (−0.008 to 0.068); both intervals included zero. The isotype term is thus a statistically supported association, not a demonstrated improvement in risk prediction.

In the pre-specified extended model additionally including serum LDH and melphalan dose (*n* = 122), the IgA estimate was similar (PFS HR 1.77, 95% CI 1.07–2.92, *p* = 0.026; OS HR 1.84, 95% CI 1.04–3.26, *p* = 0.036), while neither LDH (PFS HR 0.94 per doubling, *p* = 0.816) nor melphalan 140 mg/m^2^ (PFS HR 0.53, *p* = 0.229) showed a statistically significant association with outcome (Table S2).

### 3.3. Exploratory Analysis of t(4;14)

t(4;14) was assessed in 64 patients (32.7%) and found in 3 (4.7%), all IgG: 0 of 17 IgA patients (exact 95% CI 0–19.5%) and 3 of 47 IgG patients (6.4%; 95% CI 1.3–17.5%). No del(17p) or t(14;16) was detected, but these probes were applied to only 21 and 20 patients, so their absence carries little information. The observed t(4;14) frequency is below the 10–15% expected in unselected myeloma, for the reasons set out in Section 4.4.

Within each isotype, patients tested for t(4;14) did not differ in PFS from those who were not (IgA: median 19.7 versus 13.8 months, *p* = 0.683; IgG: 42.7 versus 28.5 months, *p* = 0.226; testing-by-subtype interaction *p* = 0.869), although testing was more frequent in later years and the point estimate favored tested patients (HR 0.77, 95% CI 0.51–1.17). Among the 61 patients without t(4;14) (17 IgA, 44 IgG), median PFS was 19.7 months (95% CI 10.4–56.6) in IgA versus 42.5 months (27.4–79.5) in IgG patients (15 and 30 events; univariable HR 2.04, 95% CI 1.08–3.86; *p* = 0.029), and median OS 70.6 months (36.5–not reached) versus not reached (94.2–not reached) (13 and 15 deaths; HR 2.97, 95% CI 1.40–6.27; *p* = 0.004). With 17 IgA patients, no adjusted model was fitted. These estimates show that the association was present in the subgroup in which the single tested lesion was absent; they do not show that it is independent of cytogenetic risk because the lesions that carry most of that risk were not assessed.

### 3.4. Combined Subtype and ISS Stratification

**Figure 2:**
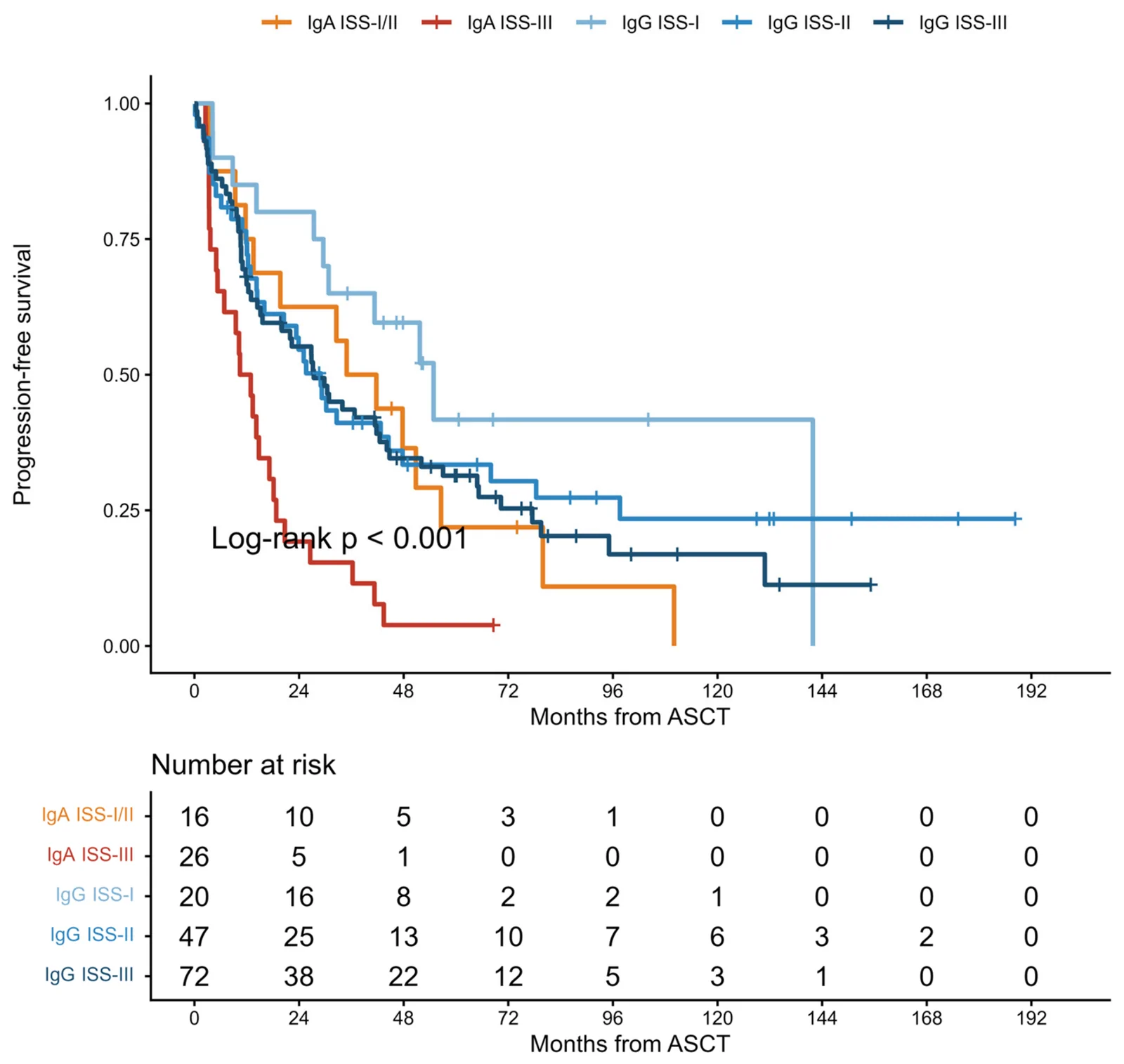
Progression-free survival across the five subtype–stage groups: IgG ISS stage I (*n* = 20), IgG ISS stage II (*n* = 47), IgG ISS stage III (*n* = 72), IgA ISS stage I/II (*n* = 16), and IgA ISS stage III (n = 26). Overall log-rank *p* < 0.001; numbers at risk are shown below the panel.

Stratifying by subtype and ISS stage produced a clear hierarchy (Table 3, Figure 2). IgG patients with stage I disease fared best (median PFS 54.9 months; 5-year PFS 41.7%, 95% CI 22.3–78.1), while IgG stage II and stage III patients clustered closely together (29.0 and 27.4 months; 5-year PFS 33.4% and 31.4%), indicating that ISS discriminated poorly within IgG disease in this cohort. By contrast, IgA patients with stage III disease had a median PFS of 11.7 months (95% CI 5.3–18.8) and a 5-year PFS of 3.8% (95% CI 0.6–26.3), an estimate resting on a single patient still at risk at 60 months and therefore imprecise. Of these 26 patients, 6 had been tested for t(4;14); none were positive. The cytogenetic risk of the group is otherwise unknown. The overall log-rank test across the five groups was significant (*p* < 0.001), but this comparison is descriptive: it does not validate a new risk classification, and the isotype-by-ISS interaction was not significant (*p* = 0.127).

### 3.5. The Lenalidomide Maintenance Era and Subgroups

Among patients transplanted from 2015 onwards, maintenance rates were similar between isotypes (IgA 76%, IgG 70%), yet median PFS remained markedly shorter for IgA patients (15.7 versus 30.5 months; *p* = 0.004; Figure S2B). In the earlier era, the difference was directionally consistent but did not reach significance in the smaller sample (14.8 versus 30.2 months; *p* = 0.122; Figure S2A). Formal interaction testing found no evidence of modification of the IgA effect by maintenance use (*p* = 0.742) or by ISS stage (*p* = 0.127); these tests have limited power in a cohort of this size, and a non-significant interaction does not show that maintenance provides no benefit in either group.

Subgroup analysis (Table 4) showed hazard ratios greater than 1.0 across all 16 subgroups examined. The point estimate reached significance among patients with ISS stage III disease (HR 2.45; *p* < 0.001), those aged under 60 years (HR 2.01; *p* = 0.007), maintenance recipients (HR 1.55; *p* = 0.034), those transplanted from 2015 onwards (HR 1.93; *p* = 0.005), those receiving full-dose melphalan (HR 1.67; *p* = 0.007), those tested for t(4;14) (HR 2.07; *p* = 0.024) and those with lambda light chains (HR 2.21; *p* = 0.005). The subgroups overlap, and the analysis is descriptive.

### 3.6. Proportional Hazards, Landmark Analyses, and the Lenalidomide Hazard Ratio

Schoenfeld residual testing indicated a violation of the proportional hazards assumption for lenalidomide maintenance (PFS *p* = 0.001; OS *p* = 0.002) and, less markedly, for age (PFS *p* = 0.012; OS *p* = 0.039), with significant global tests (PFS *p* = 0.006; OS *p* = 0.018). The isotype term itself satisfied the assumption in both models (PFS *p* = 0.460; OS *p* = 0.222; Table S5). In a sensitivity model stratified on maintenance status, the global test was no longer significant (PFS *p* = 0.276; OS *p* = 0.543) and the IgA estimate was essentially unchanged (PFS HR 1.94, 95% CI 1.20–3.14, *p* = 0.007; OS HR 1.80, 95% CI 1.06–3.04, *p* = 0.029), but stratification did not fully resolve the age finding: the age-specific test was *p* = 0.056 for PFS in the stratified model, and in the model without maintenance it was *p* = 0.005 for PFS and *p* = 0.042 for OS. We therefore allowed the age coefficient to vary with log time (Section 2.5). In the base model this gave IgA hazard ratios of 1.78 (95% CI 1.11–2.85; *p* = 0.018) for PFS and 1.78 (1.05–3.02; *p* = 0.033) for OS, and in the baseline-only model 1.74 (1.06–2.86; *p* = 0.028) and 1.63 (0.95–2.79; *p* = 0.077), respectively; the age-by-log-time terms had *p*-values between 0.021 and 0.046. Accounting for the non-proportional age effect thus moved the isotype estimates only slightly, in the same direction as the adjustment for calendar year.

**Figure 3:**
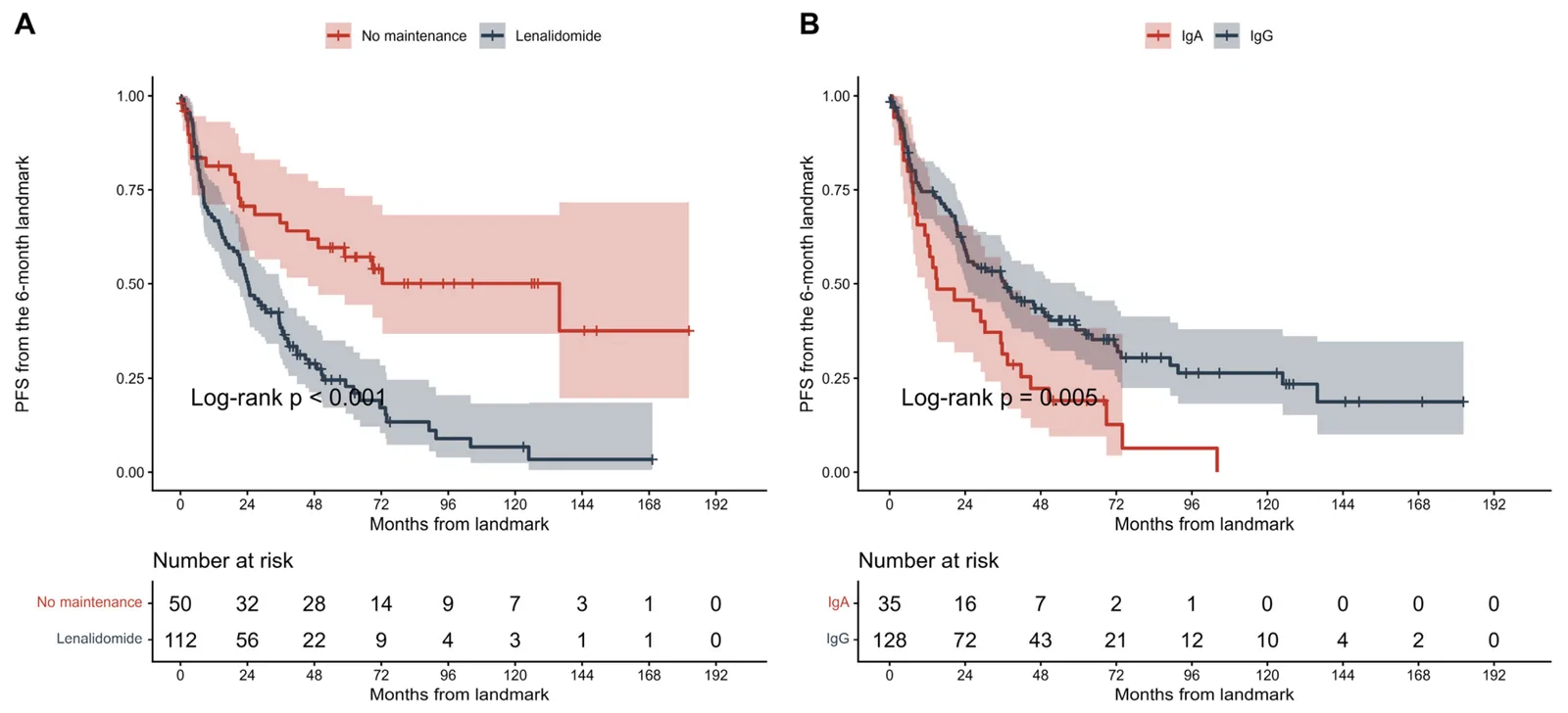
Six-month landmark analysis in all 163 eligible patients. (**A**) PFS from the landmark by maintenance status (*n* = 162: 50 without and 112 with maintenance; the patient with unknown maintenance status is excluded from this panel); the apparent advantage of the non-maintenance group may reflect incomplete documentation of progression rather than harm from maintenance (Section 3.6). (**B**) PFS from the landmark by subtype in all 163 patients (IgA *n* = 35, IgG *n* = 128; log-rank *p* = 0.005).

In the 6-month landmark population (163 eligible patients: 35 IgA, 128 IgG), median PFS from the landmark was 15.1 versus 36.5 months (31 and 83 events; log-rank *p* = 0.005; Figure 3B; Table 5). The adjusted model, fitted in the 110 complete cases among them (22 IgA, 88 IgG; 74 events, 19 in IgA patients), gave an HR of 1.72 (95% CI 0.98–3.02; *p* = 0.061). At the 12-month landmark (133 eligible: 28 IgA, 105 IgG), median PFS was 23.6 versus 43.4 months (24 and 63 events; log-rank *p* = 0.003), with an adjusted HR of 1.76 (95% CI 0.91–3.40; *p* = 0.092) in 93 complete cases (18 IgA; 58 events). Both adjusted landmark estimates are directionally consistent with the primary analysis and of similar magnitude, but their confidence intervals include unity; they are supportive but imprecise, and we do not present them as confirmation.

The hazard ratio above 1.0 for lenalidomide maintenance in the PFS models requires explanation. Unadjusted median PFS was longer in patients who did not receive maintenance (51.7 versus 25.1 months; *p* < 0.001), yet overall survival showed no statistically significant association with maintenance (median 65.6 versus 73.2 months; log-rank *p* = 0.967; adjusted HR 0.92, 95% CI 0.51–1.65, *p* = 0.778, from the base model). A discrepancy of this kind, confined to the endpoint that depends on documented progression, is consistent with differential ascertainment rather than a treatment effect. Among patients who died, a preceding progression had been recorded in 69 of 77 deaths (89.6%) in the maintenance group and in 13 of 32 deaths (40.6%) in the non-maintenance group (Fisher *p* < 0.001; Table 6). In the non-maintenance group, therefore, the PFS event was often recorded at death rather than at progression, and living patients whose progression went unrecorded were censored; both mechanisms lengthen measured PFS. We cannot exclude that some of these deaths occurred without clinical progression, and differential ascertainment is a possible explanation for the paradoxical hazard ratio rather than an established one.

Among patients who died, a progression was documented before death in 31 of 37 IgA deaths (83.8%) and 51 of 73 IgG deaths (69.9%; *p* = 0.164); within maintenance strata the proportions were 92.9% versus 87.8% among maintenance recipients and 62.5% versus 33.3% among non-recipients (8 and 24 deaths), and within maintenance-by-era strata the groups were small (for example, 4 of 5 IgA versus 4 of 9 IgG deaths among non-recipients transplanted from 2015 onwards; Table S7). These comparisons suggest a documentation difference associated with maintenance, but the absence of statistical significance in groups of this size does not establish that ascertainment was similar by isotype or era, and differences in the observed magnitude cannot be excluded. Documentation among patients who later died is, moreover, not a direct measure of the completeness or timing of progression ascertainment in the whole cohort, particularly among patients still alive. The lenalidomide covariate in our PFS models is therefore best interpreted as a marker of follow-up intensity, not as a treatment effect, and adjustment for it cannot be assumed to eliminate ascertainment bias in the isotype comparison. The IgA estimate was similar in the maintenance-stratified model, in the model without the maintenance covariate, in the era-stratified analyses, and at both landmarks, and the OS estimate, which does not depend on progression documentation, pointed in the same direction.

### 3.7. Sensitivity of the IgA Estimate to Model Specification

Table S3 collects the sensitivity models. Coding ISS stage and response depth as categorical variables gave an IgA HR of 1.78 (95% CI 1.08–2.91; *p* = 0.022) for PFS and 1.84 (1.06–3.19; *p* = 0.029) for OS; the stage coefficients were not linear (stage II 1.64, stage III 2.00 relative to stage I for PFS), but the isotype estimate was little affected. Adding light-chain type left the estimate unchanged (PFS 1.90, 1.18–3.06; OS 1.79, 1.05–3.06); the lambda light chain itself was not associated with outcome after adjustment (PFS HR 0.87, 0.55–1.37; OS 1.10, 0.65–1.84), and there was no isotype-by-light-chain interaction (*p* = 0.887).

Omitting response depth allowed 179 patients to be analyzed and gave an HR of 1.76 (1.20–2.58; *p* = 0.004) for PFS and 2.10 (1.37–3.23; *p* = 0.001) for OS; refitting the same model in the 131 complete cases gave 1.62 (1.03–2.56) and 1.67 (0.99–2.80), so the difference between the base model and the larger-sample model reflects both the covariate and the sample. Multiple imputation over all 196 patients gave 1.82 (1.25–2.66; *p* = 0.002) for PFS and 2.12 (1.39–3.23; *p* = 0.001) for OS with the base-model covariates, and 1.98 (1.36–2.88) and 2.06 (1.36–3.11) without the maintenance covariate. Patients excluded from the complete-case analysis were transplanted earlier (58.5% before 2015 versus 18.3%; *p* < 0.001) and more often received doublet induction (68.8% versus 45.0%; *p* < 0.001), but did not differ in age, sex, ISS stage, LDH, death rate or median survival (Table S6), and the univariable IgA estimate was similar in the two groups (PFS HR 1.79 in both).

Adjustment for the treatment era behaved differently. Adding induction regimen alone did not change the estimate (PFS 1.84, 1.14–2.97; OS 1.84, 1.08–3.12). Adding calendar year of transplantation, alone or with regimen, attenuated it: PFS HR 1.68 (95% CI 1.02–2.75; *p* = 0.041) and OS HR 1.72 (0.99–2.97; *p* = 0.052) with both covariates, the year coefficient being 0.94 per year (0.87–1.01) for PFS. Year and regimen were only moderately collinear (variance inflation factor 1.53). Within patients induced with bortezomib–dexamethasone (n = 59) the PFS estimate was 1.15 (0.58–2.27), and within those induced with a triplet (n = 72) it was 2.72 (1.39–5.32); the interaction was not significant (*p* = 0.162) and the subgroups are small, but we report the heterogeneity because it bears on the claim that a common backbone isolates the isotype effect (Section 4.5).

These models place the main, baseline-only analysis (Table 2A) in context. Compared with the base model, it yielded a similar PFS estimate (1.85 versus 1.87) and a smaller OS estimate (1.68 versus 1.82), with year coefficients of 0.94 for PFS (0.87–1.01) and 0.95 for OS (0.87–1.04). Once the period of treatment is taken into account, the PFS association remains of similar size, and the OS association is of uncertain magnitude: its complete-case confidence interval includes unity, while the imputed estimate, which retains the 65 patients excluded from the complete-case analysis (50 without a recorded pre-transplant response assessment and 15 with a recorded response but missing ISS stage or age), does not. Neither result settles the question on its own. Calendar year in this cohort carries induction intensity, maintenance availability, supportive care, and follow-up practice, and is a plausible confounder in its own right.

The E-value for the adjusted PFS hazard ratio was 2.46, and 1.46 for the lower confidence limit; for OS, the values were 2.39 and 1.28 (base model). An unmeasured confounder would need to be associated with both the IgA isotype and the outcome by a risk ratio of about 2.4–2.5 to fully explain the point estimate, and of about 1.3–1.5 to bring the confidence interval to the null. High-risk cytogenetic lesions with an isotype-specific prevalence could plausibly reach the latter value.

The three patients with an unusable progression date (Section 2.2) were not among the complete cases, so excluding them leaves the multivariable estimates unchanged; across the remaining 193 patients the univariable hazard ratios were 1.83 (1.28–2.63) for PFS and 2.05 (1.37–3.07) for OS, and the multiply imputed base-model estimates 1.83 (1.24–2.70) and 2.19 (1.43–3.38). When all living patients were instead assumed to be alive on 29 April 2026, the OS hazard ratio was 1.79 in the base model and 1.69 in the baseline-only model (1.82 and 1.68 with censoring at the last documented date alive); assuming survival for a further 1 to 12 months gave 1.80–1.87 and 1.69–1.71 (Table S9). These analyses hold every death and death date fixed and lengthen only the censored times of living patients, whose vital status was not re-ascertained at those dates. They show the numerical sensitivity of the fitted models to the assumption of survival to a common date; they are not additional observed follow-up and cannot establish when, or whether, vital status was verified. PFS is unaffected because living patients without progression are censored at the last recorded visit under every rule.

Measured post-progression survival was numerically shorter in IgA patients (median 18.3 versus 28.9 months; HR 1.55, 95% CI 0.99–2.43; *p* = 0.056) in the 119 patients with a documented progression. This comparison needs caution. Its estimate is imprecise, and post-progression survival is measured from the recorded progression date, so if progression is recognized later in IgA disease, the measured interval from progression to death is shortened mechanically even when the date of death and the underlying prognosis are unchanged. Shorter measured post-progression survival is therefore not, by itself, evidence that delayed recognition worsened outcomes. Median potential follow-up by reverse Kaplan–Meier was 86.2 months (95% CI 77.2–108.7) overall, 81.0 in IgA and 86.2 in IgG patients, 150.8 for transplants before 2015, and 74.7 for those from 2015 onwards.

## 4. Discussion

In this single-center cohort of 196 patients undergoing upfront ASCT, IgA myeloma was associated with inferior outcomes. In the main analysis, which used only information available at transplantation and adjusted for stage, age, depth of pre-transplant response, induction regimen, and year of transplantation, the hazard ratio was 1.85 for progression-free survival (95% CI 1.13–3.02) and 1.68 for overall survival (0.98–2.88) in complete cases, and 1.83 and 1.91 by multiple imputation. Secondary models that also included eventual maintenance receipt gave estimates of about 1.8 for both endpoints. The progression-free survival association was therefore consistent across specifications, whereas the magnitude of the overall survival association is uncertain: its complete-case confidence interval included unity and its imputed interval did not, a difference that reflects the information available and the missing-data assumptions rather than a resolution in either direction. The optimism-corrected gain in discrimination when isotype was added to a stage-based model was small, and the bootstrap interval for the apparent gain included zero. IgA patients entered transplantation with an apparent advantage: they achieved complete response before transplantation more often than IgG patients (70.6% versus 50.9%) and received maintenance at least as frequently. They nonetheless progressed and died sooner.

### 4.1. Relationship to Existing Evidence

Our observation is consistent with the largest contemporary dataset addressing this question. Bal and colleagues analyzed 8468 patients in the Flatiron Health database and found that the IgA isotype was associated with an adverse effect on overall survival that persisted after adjustment for variables known to influence outcomes, concluding that this likely reflects biology beyond the presence of established adverse prognostic factors [28]. That study, two orders of magnitude larger than ours and drawn from a more recent treatment era, reaches the same conclusion, and our data extend it specifically to the post-transplant setting.

### 4.2. Three Candidate Explanations

Three explanations for an isotype-associated survival difference merit consideration, and they are not mutually exclusive.

The first is cytogenetic enrichment. The association between IgA isotype and t(4;14) is well documented and was confirmed in the novel-agent era by Sato and colleagues, who found t(4;14) in 9 of 23 IgA patients compared with 7 of 56 IgG patients (*p* = 0.02) and showed that it remained an independent predictor of overall survival [10]. Because t(4;14) arises from class-switch recombination errors at the immunoglobulin heavy chain locus, an isotype-specific bias in the translocation repertoire is biologically coherent and would plausibly extend to lesions our panel did not assess. Our own data cannot adjudicate this: no IgA patient among the 17 tested carried t(4;14). The exact upper confidence limit of 19.5% is below the 39.1% (9 of 23) reported by Sato, so the interval does not include that frequency. The discrepancy is more plausibly explained by the lower sensitivity of our assay, performed on unselected marrow without plasma cell enrichment (Section 4.4), and by selection of the patients who were tested, than by a genuinely low prevalence of t(4;14) in IgA disease; with one probe applied to a third of the cohort, 1q21 gain and del(1p32) not assessed, and del(17p) assessed in a tenth, this is weak evidence of absence. The E-value analysis (Section 3.7) quantifies this point: a lesion twice as common in IgA disease and carrying a modest hazard would shift the confidence interval toward the null.

The second is measurement. IgA monoclonal proteins co-migrate with the beta fraction on serum protein electrophoresis and are quantified unreliably at low concentrations. Visram and colleagues studied 491 patients with IgA myeloma and showed that the electrophoretic monoclonal protein nadir preceded the nephelometric quantitative IgA nadir by a median of 41 days, that response assessment by standard IMWG criteria may be falsely reassuring, and that progression is detected later than with quantitative IgA [12]. The Hevylite assay, which quantifies heavy and light chain pairs separately, produces measurable results in IgA sera where electrophoresis cannot [29], although a recent analysis within the GMMG-MM5 trial did not support its routine use for response assessment [30]. Three distinct mechanisms follow, each acting in a different direction. First, the higher pre-transplant complete response rate we observed in IgA patients may be partly artifactual, so that adjustment for response depth is adjustment for a differentially misclassified covariate; this could bias the isotype estimate in either direction. Second, if progression is detected later in IgA disease, the recorded PFS event occurs later, and measured PFS is lengthened rather than shortened; delayed detection alone, therefore, cannot explain the inferior PFS we observed. This mechanism, acting alone, would bias the PFS comparison toward the null, but it does not establish the net direction of bias because documentation, treatment, covariate misclassification, and selection may also differ between the groups. Third, later clinical recognition could delay salvage therapy and worsen OS. Measured post-progression survival was numerically shorter in IgA patients (Section 3.7), but a later recorded progression date mechanically shortens that interval, so the finding cannot distinguish a real effect of delayed salvage from an artifact of timing; the treatment-mediated explanation remains untested. The measurement hypothesis thus bears on how the response and progression variables should be read. Delayed detection of progression alone would lengthen measured PFS, but the combined effect of measurement, documentation, and selection on the isotype estimate cannot be determined from these data.

The third is humoral immune reconstitution. Immunoparesis is an established adverse prognostic factor in myeloma, and recovery of uninvolved immunoglobulins after autologous transplantation is associated with better outcomes [11]; Gao and colleagues showed that failure of immunoglobulin recovery 1 year after ASCT was independently associated with inferior overall survival [31]. Whether immunoparesis is deeper or more persistent in IgA than in IgG myeloma has not, to our knowledge, been tested directly. Serial immunoglobulin levels were not recorded in our registry, so we could not test them here; we regard this as the most important gap to close in a follow-up study. Because post-transplant immune reconstitution is measurable and modifiable, this is the most tractable of the three hypotheses, and it applies specifically to the autologous setting.

### 4.3. The IgA and ISS Stage III Subgroup

The combination of IgA subtype with ISS stage III identified a group with a median PFS of 11.7 months and a 5-year PFS of 3.8%, the latter with a wide confidence interval (0.6–26.3%) and a single patient at risk at 5 years. Only 6 of these 26 patients had been tested for t(4;14); none were positive, and none had been assessed for del(17p) with a result. The group is therefore incompletely characterized for cytogenetic risk, and we cannot say whether current cytogenetic staging would classify it as high-risk. The data show that, within a cohort staged by ISS alone, IgA stage III patients fared worse than IgG stage III patients. The subgroup is small and the estimate imprecise, the five-group comparison is descriptive, and the isotype-by-stage interaction was not significant, so we present this as an observation for larger cohorts to examine rather than as a proposed risk category.

### 4.4. The Low Prevalence of t(4;14) and the Limits of the Cytogenetic Data

t(4;14) was found in 3 of 64 tested patients (4.7%), below the 10–15% expected, and no del(17p) or t(14;16) was detected among the 21 and 20 patients assessed. Three factors contribute. Most importantly, iFISH was performed on an unselected bone marrow aspirate without CD138 immunomagnetic enrichment; sensitivity falls when the plasma cell fraction is low, and abnormalities confined to a minority clone are missed. Second, the panel comprised only three probes, applied unevenly, so 1q21 gain and amplification, del(1p32) and biallelic TP53 events could not contribute, and del(17p) itself was assessed in a tenth of the cohort; contemporary cytogenetic prognostic indices weigh several of these lesions heavily [17,32]. Third, testing was performed in a minority of patients and more often in later years. For these reasons, we make no claim about the cytogenetic risk profile of either isotype group, and the t(4;14)-negative subgroup should be understood as free of one detectable abnormality on a limited assay rather than as genomically standard-risk.

### 4.5. Interpreting a Cohort Treated Before Routine Anti-CD38 Use

Every patient in this series was treated with a proteasome-inhibitor backbone; 196 received a daratumumab-containing regimen before transplantation. In well-resourced health systems, contemporary practice differs, and quadruplet induction is now standard for transplant-eligible patients; in the PERSEUS trial, daratumumab added to bortezomib, lenalidomide, and dexamethasone produced markedly higher rates of minimal residual disease negativity and improved progression-free survival [33]. Two consequences follow, and they point in different directions.

The first constraint generalizability. Our absolute survival estimates describe bortezomib-era practice and are not contemporary benchmarks for centers using quadruplet induction.

The second is analytical. A cohort treated on a common backbone reduces the regimen heterogeneity that characterizes the present transition, during which quadruplet, triplet, and doublet induction coexist, and access varies widely between and within health systems. In such mixed cohorts, treatment intensity is strongly correlated with calendar time, center, and reimbursement status, so any isotype estimate depends on adjustment for a covariate that is itself a proxy for era and access. Our cohort is not free of this problem. Half the patients received a doublet and half a triplet; the mix changed over time, and when calendar year was added to the model, the IgA estimate was about 1.7–1.9, and for overall survival, its complete-case confidence interval crossed unity, while the estimate also differed between doublet- and triplet-induced patients, without a significant interaction. Calendar year in this cohort carries induction intensity, maintenance availability, supportive care, and, given the ascertainment pattern described in Section 3.6, follow-up intensity. That is why the model adjusted for calendar year is our main basis for interpretation. Its lower confidence limits in complete cases lie above (PFS) or just below (OS), no effect. Nor can we claim that the backbone isolates the isotype effect. What the design offers is a narrower range of treatment heterogeneity than a cohort spanning the anti-CD38 transition, not its absence.

Whether the association persists under quadruplet therapy is an open question that our data cannot settle, and two considerations pull in opposite directions. The Flatiron analysis of 8468 patients found the IgA disadvantage persisted through the period in which proteasome inhibitors and immunomodulatory drugs became standard [28]; and if part of the effect arises from the measurement problem described in Section 4.2, more effective therapy will not remove it, because the difficulty lies in how response and progression are detected in IgA disease rather than in how well the disease responds. Against this, a deeper and more uniform starting point could compress isotype-associated differences. The present cohort cannot determine the magnitude of the association under contemporary treatment or whether it persists; that question remains untested. We therefore suggest that immunoglobulin isotype be pre-specified as a stratification variable in trials and registries reading out under quadruplet induction, rather than assuming the association will either persist or disappear.

Finally, the question the study raises is most immediate where anti-CD38 antibodies are not routinely available in the first line, which remains the case across much of the world. In those settings, the treatment backbone resembles that described here; expanded cytogenetic panels and serial MRD monitoring are often unavailable, and a prognostic marker obtained as part of routine diagnostic workup has practical value.

### 4.6. Limitations

This study has substantial limitations that constrain the strength of its conclusions.

The cytogenetic assessment is the most important. t(4;14) was assessed in a third of patients and del(17p) and t(14;16) in a tenth; the panel omitted 1q21 gain and amplification, del(1p32) and biallelic TP53 inactivation, and hybridization was performed without plasma cell enrichment. We cannot, therefore, exclude that IgA myeloma in this cohort was enriched for high-risk lesions we did not measure, and unmeasured cytogenetic confounding remains a viable alternative to the hypothesis of isotype-intrinsic biology; the E-values indicate that a confounder of moderate strength would suffice to bring the confidence interval to the null. We have deliberately avoided describing the association as cytogenetically occult or as independent of cytogenetic risk for this reason.

Recording progression is a further limitation. As set out in Section 3.6, a progression was recorded before death in 89.6% of maintenance recipients who died and in 40.6% of non-recipients who died. These are observed proportions among deaths, not estimates of the proportion of true progressions that were captured, because some deaths may have occurred without a preceding progression. Where progressions went unrecorded, measured progression-free survival would be lengthened, and living patients without a recorded progression were censored at the last recorded hospital visit, which the registry does not identify as a disease assessment. The comparison by maintenance status does not prove that under-documentation is the only explanation of the paradoxical lenalidomide hazard ratio; it is a possible explanation. These data describe patients who died and are not a direct estimate of ascertainment among survivors; comparisons by isotype in small groups cannot exclude a difference in ascertainment between IgA and IgG disease. Adjustment for maintenance, therefore, cannot be assumed to remove ascertainment bias from the isotype comparison. The 3-monthly assessment schedule was the institutional intention; adherence to it could not be verified from the registry. Overall survival, which does not depend on progression documentation, is the more reliable endpoint in this dataset and shows the same direction, although it was also the endpoint most sensitive to adjustment for calendar year and to the handling of missing data.

Maintenance was recorded as eventual receipt, without a start date. It is therefore a post-baseline exposure, and its inclusion as a covariate can introduce immortal-time bias; landmark analyses reduce but do not eliminate this bias because they still classify by eventual receipt. We have addressed it by reporting models without the covariate and by reading the covariate as a marker of follow-up intensity, which does not remove the temporal problem; a time-dependent analysis would require dates we do not have. A non-significant interaction in a cohort of this size does not show whether maintenance benefits the two groups equally or unequally. What can be said is that an IgA-associated difference remained observable among maintenance recipients.

Pre-ASCT response depth, which we use as an adjustment variable, may be differentially misclassified by isotype for the measurement reasons discussed in Section 4.2. Models without this covariate yielded similar isotype estimates, but residual misclassification remains possible. The complete-case analysis excluded a third of patients, predominantly from the earlier period; the imputation analysis gave similar estimates but rests on its own untestable assumptions. Several variables were not held in the transplant registry and could not be recovered for this analysis. We have no measure of post-transplant depth of response, so we cannot determine whether the IgA disadvantage reflects a failure to deepen response after transplantation or early relapse despite a deep response; these are mechanistically different, and distinguishing them would require the day-100 and best post-transplant response data we lack. Minimal residual disease was not routinely assessed during most of the study period. Serial immunoglobulin levels, and hence immunoparesis and its recovery, were not recorded, so the third hypothesis in Section 4.2 could not be tested. Serum albumin and β2-microglobulin were included in the analysis only at the ISS stage, so we cannot say which component drives the stage effect. The mode and timing of progression detection, as well as the number of prior treatment lines, were not recorded. Three recorded progression dates could not be reconciled with the dates of transplantation or death and were treated as unusable, which did not affect the multivariable estimates. No dated record of a cohort-wide vital-status query was retained, so living patients were censored at the last documented date alive; for 15 of them, this was more than a year before the end of follow-up. Carrying all living patients to a common date, under the assumption that they were alive, changed the OS estimates little (Section 3.7), but that analysis cannot establish their vital status. Post-transplant salvage therapy, including any later anti-CD38 exposure, was likewise not captured, so we cannot examine whether access to modern agents at relapse modified the isotype effect on overall survival.

Treatment was also heterogeneous within the cohort, as discussed in Section 4.5, and the attenuation of the estimate when calendar year was added is a genuine uncertainty rather than a technicality. Serum LDH was measured before conditioning rather than at diagnosis, so our null result for LDH does not test the R-ISS criterion. The cohort excludes light-chain-only disease, the other isotype group with an adverse reputation, and is therefore not representative of all transplant-eligible myeloma. Finally, the single-center retrospective design and the modest number of IgA patients limit power in subgroup analyses and preclude any causal interpretation. Validation in registry-scale cohorts with complete cytogenetic characterization, such as CIBMTR, EBMT, or CoMMpass, is the appropriate confirmatory step.

### 4.7. Clinical Perspective

Immunoglobulin isotype is determined at diagnosis in every patient with intact-immunoglobulin myeloma as part of routine work-up, at no additional cost, and is reproducible across laboratories. In settings where expanded cytogenetic panels and serial MRD monitoring are unavailable or inconsistently accessible, an isotype-based signal of higher risk would be of practical value. Our data are insufficient to recommend that isotype guide treatment decisions or be added to existing risk models, and we do not propose either. They are sufficient to argue that isotype should be recorded, reported, and tested as a stratification variable in prospective studies, and that its exclusion from current risk models deserves formal re-examination in cohorts where full genomic characterization makes it possible to distinguish an isotype effect from cytogenetic confounding, and where external validation of any incremental prognostic value is possible.

## 5. Conclusions

In a transplant cohort treated on a proteasome-inhibitor backbone, before anti-CD38 antibodies entered routine first-line use, IgA myeloma was associated with inferior progression-free survival after adjustment for information available at transplantation, including induction regimen and year of transplantation. The overall survival association was in the same direction but of uncertain magnitude once the treatment period was accounted for: its confidence interval included unity in complete cases but not after multiple imputation. Secondary models that included maintenance therapy gave similar or larger estimates. The combination of IgA subtype with ISS stage III identified a small subgroup with very poor outcomes whose cytogenetic risk is unknown. An IgA-associated difference remained observable among maintenance recipients and in the later treatment era, and landmark estimates were directionally consistent but imprecise. Because the recording of progression differed between patient groups, overall survival is the more reliable of our endpoints, and it shows the same direction. Because our cytogenetic data were too incomplete to assess independence from genomic risk, and because IgA paraprotein measurement is itself unreliable, these findings are hypothesis-generating. They apply most directly to the many health systems in which first-line anti-CD38 therapy is not routinely available, and they identify immunoglobulin isotype as a variable worth pre-specifying in registry-scale cohorts with complete genomic characterization and in patients treated with quadruplet induction.

## Figures and Tables

**Table 1:** Baseline characteristics of the IgA and IgG myeloma cohort (*n* = 196).

Variable	All (*n* = 196)	IgA (*n* = 47)	IgG (*n* = 149)	*p*	SMD
Age at ASCT, median (IQR), years	58.6 (52.9–63.7)	60.1 (52.9–64.4)	58.4 (52.9–63.4)	0.793	0.02
Female sex, n/N (%)	91/196 (46.4)	21/47 (44.7)	70/149 (47.0)	0.867	0.05
Light chain: kappa, n/N (%)	129/196 (65.8)	24/47 (51.1)	105/149 (70.5)	**0.021**	0.41
Light chain: lambda, n/N (%)	67/196 (34.2)	23/47 (48.9)	44/149 (29.5)	—	0.41
Diagnosis to ASCT, median (IQR), months	12.7 (9.5–17.6)	13.4 (9.6–28.3)	12.6 (9.5–17.2)	0.423	0.02
ISS stage I, n/N (%)	23/181 (12.7)	3/42 (7.1)	20/139 (14.4)	0.364	0.24
ISS stage II, n/N (%)	60/181 (33.1)	13/42 (31.0)	47/139 (33.8)	—	0.06
ISS stage III, n/N (%)	98/181 (54.1)	26/42 (61.9)	72/139 (51.8)	—	0.21
Induction: bortezomib–dexamethasone	103/195 (52.8)	25/46 (54.3)	78/149 (52.3)	0.665	0.04
Induction: VCD	25/195 (12.8)	4/46 (8.7)	21/149 (14.1)	—	0.17
Induction: VRD or other IMiD triplet	67/195 (34.4)	17/46 (37.0)	50/149 (33.6)	—	0.07
Any anti-CD38 antibody before ASCT	1/196 (0.5)	0/47 (0.0)	1/149 (0.7)	—	0.12
Pre-ASCT complete response	81/146 (55.5)	24/34 (70.6)	57/112 (50.9)	**0.030** ‡	0.41
Pre-ASCT VGPR	48/146 (32.9)	5/34 (14.7)	43/112 (38.4)	—	0.56
Pre-ASCT partial response	17/146 (11.6)	5/34 (14.7)	12/112 (10.7)	—	0.12
Melphalan 200 mg/m^2^	185/194 (95.4)	44/45 (97.8)	141/149 (94.6)	0.688	0.17
Melphalan 140 mg/m^2^	9/194 (4.6)	1/45 (2.2)	8/149 (5.4)	—	0.17
Serum LDH, median (IQR), U/L	190 (161–227)	212 (169–251)	185 (155–217)	**0.027**	0.42
LDH available, n	174	38	136	—	—
Creatinine, median (IQR), mg/dL	0.74 (0.66–0.91)	0.73 (0.66–0.82)	0.74 (0.66–0.93)	0.456	0.06
CD34+ dose, median (IQR), ×10^6^/kg	5.0 (4.1–6.8)	5.0 (4.0–6.6)	5.0 (4.2–6.8)	0.451	0.08
Neutrophil engraftment, median (IQR), days	11 (11–12)	11 (11–12)	11 (11–12)	0.975	0.14
Lenalidomide maintenance	132/195 (67.7)	36/46 (78.3)	96/149 (64.4)	0.104	0.31
ASCT from 2015 onwards	134/196 (68.4)	30/47 (63.8)	104/149 (69.8)	0.474	0.13
t(4;14) tested by iFISH	64/196 (32.7)	17/47 (36.2)	47/149 (31.5)	0.594	0.10
t(4;14) positive (of tested)	3/64 (4.7)	0/17 (0.0)	3/47 (6.4)	—	—
del(17p) tested by iFISH	21/196 (10.7)	2/47 (4.3)	19/149 (12.8)	—	—
t(14;16) tested by iFISH	20/196 (10.2)	2/47 (4.3)	18/149 (12.1)	—	—
Observed follow-up (to death or last documented date alive), median (IQR), months	47.7 (23.8–80.1)	43.2 (18.8–74.4)	50.5 (26.8–86.5)	—	—
Deaths	110/196 (56.1)	37/47 (78.7)	73/149 (49.0)	**<0.001**	—
Progression or death	147/196 (75.0)	43/47 (91.5)	104/149 (69.8)	**0.003**	—

ASCT, autologous stem cell transplantation; IMiD, immunomodulatory drug; iFISH, interphase fluorescence in situ hybridization; IQR, interquartile range; ISS, International Staging System; LDH, lactate dehydrogenase; SMD, absolute standardized mean difference between IgA and IgG (continuous variables on the pooled standard deviation; each level of a categorical variable as a binary indicator); VCD, bortezomib–cyclophosphamide–dexamethasone; VGPR, very good partial response; VRD, bortezomib–lenalidomide–dexamethasone. Each variable is presented over its own evaluable denominator. *p*-values: Wilcoxon rank-sum (continuous), chi-square or Fisher’s exact (categorical); a single *p*-value is given for each multi-level variable. ‡ Omnibus test across CR, VGPR and PR; the 2 × 2 comparison of CR versus less than CR gives *p* = 0.050. Bold denotes *p* < 0.05. Serum LDH was measured before conditioning and after completion of induction. No patient tested for del(17p) or t(14;16) carried the abnormality. Observed follow-up is the time from ASCT to death or to the last documented date of survival and is shorter in the IgA group because more IgA patients died; potential follow-up, estimated by the reverse Kaplan–Meier method, is given in Section 3.7.

**Table 2:** Cox regression for progression-free and overall survival. (**A**) Main analysis: baseline-only model. (**B**) Secondary analyses: univariable estimates and the pre-specified base model including maintenance.

(**A**)					
**Variable**		**PFS, Complete Cases (*n* = 131), HR (95% CI)**	**OS, Complete Cases (*n* = 131), HR (95% CI)**	**PFS, Multiple Imputation (*n* = 196), HR (95% CI)**	**OS, Multiple Imputation (*n* = 196), HR (95% CI)**
IgA subtype (vs. IgG)		1.85 (1.13–3.02) *p* = 0.015	1.68 (0.98–2.88) *p* = 0.058	1.83 (1.22–2.73) *p* = 0.004	1.91 (1.24–2.94) *p* = 0.004
ISS stage (per stage)		1.36 (1.00–1.85) *p* = 0.049	1.42 (0.98–2.06) *p* = 0.065	1.34 (1.03–1.74) *p* = 0.031	1.45 (1.06–1.99) *p* = 0.020
Age (per year)		1.01 (0.98–1.04) *p* = 0.505	1.04 (1.01–1.07) *p* = 0.022	1.00 (0.98–1.02) *p* = 0.946	1.02 (1.00–1.05) *p* = 0.075
Pre-ASCT response (per category)		1.46 (1.06–2.01) *p* = 0.021	1.35 (0.94–1.95) *p* = 0.107	1.23 (0.93–1.64) *p* = 0.150	1.16 (0.84–1.59) *p* = 0.356
Induction: VCD (vs. bortezomib–dexamethasone)		0.98 (0.42–2.31) *p* = 0.961	0.48 (0.13–1.74) *p* = 0.264	1.08 (0.56–2.10) *p* = 0.816	0.68 (0.27–1.67) *p* = 0.390
Induction: VRD or other IMiD triplet (vs. bortezomib–dexamethasone)		1.22 (0.73–2.04) *p* = 0.449	1.01 (0.57–1.78) *p* = 0.971	1.19 (0.75–1.90) *p* = 0.458	1.10 (0.66–1.82) *p* = 0.718
Year of ASCT (per year)		0.94 (0.87–1.01) *p* = 0.106	0.95 (0.87–1.04) *p* = 0.310	0.95 (0.89–1.01) *p* = 0.077	0.94 (0.88–1.01) *p* = 0.117
(**B**)					
**Variable**	**n**	**Univariable PFS, HR (95% CI)**	**Base Model PFS, HR (95% CI)**	**Univariable OS, HR (95% CI)**	**Base Model OS, HR (95% CI)**
IgA subtype (vs. IgG)	196	1.82 (1.27–2.60) *p* = 0.001	1.87 (1.17–3.01) *p* = 0.010	1.98 (1.33–2.96) *p* < 0.001	1.82 (1.07–3.09) *p* = 0.026
Lambda light chain (vs. kappa)	196	1.08 (0.77–1.51) *p* = 0.670	—	1.38 (0.94–2.04) *p* = 0.101	—
ISS stage (per stage)	181	1.40 (1.09–1.79) *p* = 0.008	1.35 (0.99–1.82) *p* = 0.054	1.49 (1.10–2.01) *p* = 0.010	1.40 (0.97–2.02) *p* = 0.071
Age (per year)	195	1.00 (0.98–1.02) *p* = 0.874	1.00 (0.97–1.03) *p* = 0.830	1.02 (0.99–1.04) *p* = 0.205	1.04 (1.01–1.08) *p* = 0.021
Female sex	196	0.83 (0.60–1.15) *p* = 0.267	—	0.81 (0.56–1.19) *p* = 0.287	—
Diagnosis to ASCT (per month)	193	1.00 (1.00–1.01) *p* = 0.445	—	1.00 (1.00–1.01) *p* = 0.420	—
Pre-ASCT response (per category)	146	1.22 (0.92–1.62) *p* = 0.159	1.48 (1.09–2.02) *p* = 0.013	1.19 (0.87–1.62) *p* = 0.274	1.46 (1.03–2.08) *p* = 0.035
Melphalan 140 vs. 200 mg/m^2^	194	0.40 (0.15–1.09) *p* = 0.072	—	0.50 (0.16–1.58) *p* = 0.236	—
Serum LDH (per doubling)	174	1.03 (0.68–1.55) *p* = 0.903	—	0.87 (0.53–1.44) *p* = 0.596	—
Lenalidomide maintenance †	195	1.91 (1.30–2.81) *p* = 0.001	1.84 (1.08–3.13) *p* = 0.024	0.99 (0.66–1.50) *p* = 0.967	0.92 (0.51–1.65) *p* = 0.778
ASCT from 2015 onwards	196	0.87 (0.62–1.23) *p* = 0.427	—	0.82 (0.56–1.23) *p* = 0.340	—

The baseline-only model contains only information available at transplantation and no maintenance covariate. It was introduced during peer review and was not pre-specified; it is the main basis for interpretation (Section 2.5). Complete cases: PFS 95 events (27 in IgA patients), C = 0.631; OS 69 events (22 in IgA patients), C = 0.643. Multiple imputation: 20 imputed datasets, 147 PFS events, and 110 deaths, estimates combined by Rubin’s rules (Section 2.5). Pre-ASCT response is ordinal (CR = 1, VGPR = 2, PR = 3), so a hazard ratio above 1 denotes a worse outcome with shallower response. The complete-case and imputed OS estimates are both reported; the difference between them reflects the information available and the missing-data assumptions. Univariable estimates use the full cohort with the evaluable denominator shown (PFS events: 147 overall, 43 IgA; OS events: 110 overall, 37 IgA); base-model estimates use the 131 complete cases (PFS 95 events, 27 in IgA patients, C = 0.619; OS 69 events, 22 in IgA patients, C = 0.622). Pre-ASCT response is ordinal (CR = 1, VGPR = 2, PR = 3), so a hazard ratio above 1 denotes a worse outcome with shallower response. † Maintenance is a post-baseline exposure recorded as eventual receipt; the lenalidomide hazard ratio above 1 for PFS may reflect differential ascertainment of progression rather than harm; the covariate is best read as a marker of follow-up intensity and must not be read as a causal treatment estimate (Section 3.6). The model without this covariate is given in the text and in Table S3.

**Table 3:** Progression-free survival across subtype and ISS strata.

Subgroup	n	Events	Median PFS (95% CI), Months	5-Year PFS, % (95% CI)	At Risk at 60 Months
IgG ISS stage I	20	11	54.9 (30.8–NR)	41.7 (22.3–78.1)	4
IgG ISS stage II	47	33	29.0 (16.0–68.0)	33.4 (21.9–50.9)	12
IgG ISS stage III	72	55	27.4 (15.6–44.1)	31.4 (22.1–44.6)	18
IgA ISS stage I/II	16	14	38.3 (13.5–NR)	21.9 (8.3–57.9)	3
IgA ISS stage III	26	25	11.7 (5.3–18.8)	3.8 (0.6–26.3)	1

NR, not reached. Overall log-rank *p* < 0.001; isotype-by-ISS interaction *p* = 0.127. IgA stages I and II are combined because only 3 IgA patients had stage I disease. Fifteen patients with missing ISS stage are not represented. Five-year estimates with fewer than 5 patients at risk are imprecise and should not be read as transportable risk estimates.

**Table 4:** Subgroup analysis of IgA versus IgG for progression-free survival.

Subgroup	n IgA	n IgG	HR (95% CI)	*p*
Overall	47	149	1.82 (1.27–2.60)	0.001
ISS stage I ‡	3	20	1.36 (0.29–6.44)	0.697
ISS stage II	13	47	1.31 (0.67–2.54)	0.427
ISS stage III	26	72	2.45 (1.49–4.01)	<0.001
Pre-ASCT complete response	24	57	1.70 (0.99–2.92)	0.056
Pre-ASCT VGPR ‡	5	43	1.77 (0.62–5.10)	0.290
Pre-ASCT partial response ‡	5	12	4.30 (1.12–16.50)	0.034
Lenalidomide maintenance	36	96	1.55 (1.03–2.33)	0.034
No maintenance	10	53	2.07 (0.93–4.60)	0.074
Age under 60 years	23	85	2.01 (1.21–3.36)	0.007
Age 60 years or older	24	63	1.57 (0.94–2.62)	0.083
ASCT from 2015 onwards	30	104	1.93 (1.22–3.05)	0.005
ASCT before 2015	17	45	1.59 (0.88–2.85)	0.123
Melphalan 200 mg/m^2^	44	141	1.67 (1.15–2.41)	0.007
t(4;14) tested	17	47	2.07 (1.10–3.89)	0.024
Kappa light chain	24	105	1.54 (0.94–2.51)	0.086
Lambda light chain	23	44	2.21 (1.27–3.84)	0.005

Univariable Cox model within each subgroup; IgA is the exposure and IgG the reference. ‡ Fewer than 8 IgA patients; interpret with caution. Interaction tests: IgA by maintenance *p* = 0.742; IgA by ISS stage *p* = 0.127; IgA by light chain *p* = 0.887. Subgroups overlap; the analysis is exploratory, and *p*-values are uncorrected.

**Table 5:** Landmark and sensitivity estimates for the IgA effect on progression-free survival, and documentation of progression before death.

Analysis	Estimate	Reading
IgA HR, baseline-only model with induction regimen and ASCT year, PFS, complete cases (*n* = 131)	1.85 (1.13–3.02), *p* = 0.015	Main analysis (Table 2A)
IgA HR, baseline-only model, PFS, multiple imputation (*n* = 196)	1.83 (1.22–2.73), *p* = 0.004	Main analysis, all patients
IgA HR, complete-case base model with maintenance, PFS (*n* = 131)	1.87 (1.17–3.01), *p* = 0.010	Secondary; pre-specified (Table 2B)
IgA HR, model without maintenance covariate, PFS (*n* = 131)	2.06 (1.29–3.30), *p* = 0.003	Information available at ASCT only; no treatment-period adjustment
IgA HR, maintenance-stratified, PFS (*n* = 131)	1.94 (1.20–3.14), *p* = 0.007	Addresses PH violation
IgA HR, 6-month landmark, PFS (Cox *n* = 110)	1.72 (0.98–3.02), *p* = 0.061	Directionally consistent; imprecise
IgA HR, 12-month landmark, PFS (Cox *n* = 93)	1.76 (0.91–3.40), *p* = 0.092	Directionally consistent; imprecise
IgA median PFS, 6-month landmark (*n* = 163)	15.1 vs. 36.5 months, *p* = 0.005	All eligible patients
IgA median PFS, 12-month landmark (*n* = 133)	23.6 vs. 43.4 months, *p* = 0.003	All eligible patients
IgA HR, base model, multiple imputation, PFS (*n* = 196)	1.82 (1.25–2.66), *p* = 0.002	Sensitivity to complete-case restriction
PFS by maintenance (unadjusted)	51.7 vs. 25.1 months, *p* < 0.001	Direction inconsistent with OS
OS by maintenance (unadjusted)	65.6 vs. 73.2 months, *p* = 0.967	No statistically significant association
OS by maintenance (adjusted, base model)	HR 0.92 (0.51–1.65), *p* = 0.778	No statistically significant association; wide CI
Progression recorded before death, maintenance	69/77 (89.6%)	Observed proportion among deaths
Progression recorded before death, no maintenance	13/32 (40.6%)	Observed proportion among deaths; *p* < 0.001 versus maintenance

PH, proportional hazards. Landmark analyses retain patients alive and progression-free at the landmark time, reset survival time to zero at that point, and exclude earlier events from both numerator and denominator; Kaplan–Meier estimates use all eligible patients, and Cox models the complete cases among them. The final two rows give the proportion of deaths preceded by a recorded progression. They describe recorded progression among patients who died and are not direct estimates of the completeness of ascertainment, because some deaths may have occurred without a preceding progression. The difference between them is a possible explanation of the paradoxical lenalidomide hazard ratio in the PFS models; they do not exclude additional variation in ascertainment by isotype or era. Further sensitivity models are given in Table S3.

**Table 6:** First progression-free survival event by isotype and maintenance status, and documentation of progression before death.

Group	n	Documented Progression	Death Without Documented Progression	Censored Alive	Deaths	Progression Documented Before Death, n/Deaths (%)
IgA, maintenance	36	32	2	2	28	26/28 (92.9)
IgA, no maintenance	10	5	3	2	8	5/8 (62.5)
IgA, maintenance unknown	1	0	1	0	1	0/1
IgA, all	47	37	6	4	37	31/37 (83.8)
IgG, maintenance	96	70	6	20	49	43/49 (87.8)
IgG, no maintenance	53	12	16	25	24	8/24 (33.3)
IgG, all	149	82	22	45	73	51/73 (69.9)
All, maintenance	132	102	8	22	77	69/77 (89.6)
All, no maintenance	63	17	19	27	32	13/32 (40.6)
All patients	196	119	28	49	110	82/110 (74.5)

A death without a documented preceding progression is counted as a PFS event at the date of death (Section 2.2). All columns are derived from the same finalized first-event classification, so that in every row, PFS events = documented progression + death without documented progression, and deaths = deaths preceded by documented progression + deaths without documented progression. The latter category includes the three patients whose recorded progression date fell before transplantation or after death, and one IgA patient whose relapse and maintenance status were not recorded (shown as maintenance unknown). Progression documented before death: maintenance versus no maintenance, Fisher *p* < 0.001; IgA versus IgG, *p* = 0.164. These proportions concern patients who died and do not directly measure ascertainment among survivors.

## Data Availability

The data presented in this study are available on request from the corresponding author due to institutional privacy policies and the conditions of the ethics approval, which do not permit public deposition of patient-level data. The complete analysis code is provided as Supplementary Material. The de-identified analysis dataset from which every reported estimate is regenerated is available from the corresponding author upon reasonable request, in accordance with the ethics approval.

## Supplementary Materials

The following supporting information can be downloaded at https://www.mdpi.com/article/10.3390/cancers18193178/s1: Figure S1, patient selection flow chart; Figure S2, era-stratified progression-free survival; Table S1, missing covariate data and the complete-case restriction; Table S2, extended multivariable Cox models including serum LDH and melphalan dose; Table S3, sensitivity models for the IgA effect and bootstrap validation of the concordance index; Table S4, characterization of excluded registry records; Table S5, covariate-specific Schoenfeld tests; Table S6, comparison of patients included in and excluded from the complete-case analysis; Table S7, documentation of progression before death by isotype within maintenance and treatment-era strata; Table S8, iFISH results by probe and isotype (positive, negative, not performed, missing); Table S9, sensitivity of the overall survival estimates to the censoring date of living patients; the completed STROBE checklist; the analysis code (reproducibility package).

## Abbreviations

ASCT, autologous stem cell transplantation; CI, confidence interval; CR, complete response; ECOG, Eastern Cooperative Oncology Group; HR, hazard ratio; iFISH, interphase fluorescence in situ hybridization; IMiD, immunomodulatory drug; IMWG, International Myeloma Working Group; IQR, interquartile range; ISS, International Staging System; LDH, lactate dehydrogenase; MRD, measurable residual disease; OS, overall survival; PFS, progression-free survival; PH, proportional hazards; PR, partial response; R-ISS, Revised International Staging System; SMD, standardized mean difference; VCD, bortezomib–cyclophosphamide–dexamethasone; VGPR, very good partial response; VRD, bortezomib–lenalidomide–dexamethasone.
