# Supplementary material for: Immunoglobulin A Subtype and Survival After Upfront Autologous Stem Cell Transplantation in Multiple Myeloma: A Real-World Cohort from the Bortezomib Era
Source: Cancers (Basel). 2026 Oct 2;18(19):3178. doi: 10.3390/cancers18193178 (PMC13664181; doi:10.3390/cancers18193178)
Supplement: Supplementary file 1 [file cancers-18-03178-s001.zip › cancers-4560415-supplementary.pdf]

Supplementary Materials

Immunoglobulin A Subtype and Survival After Upfront Autologous Stem Cell Transplantation in Multiple Myeloma: A Real-World Cohort from the Bortezomib Era

Cancers, manuscript cancers-4560415, revision 3

Contents: Figure S1, patient selection flow chart; Figure S2, era-stratified progression-free survival; Table S1, missing covariate data and the complete-case restriction; Table S2, extended multivariable Cox models including serum LDH and melphalan dose; Table S3, sensitivity models for the IgA effect and bootstrap validation of the concordance index; Table S4, characterization of excluded registry records; Table S5, covariate-specific Schoenfeld tests; Table S6, comparison of patients included in and excluded from the complete-case analysis; Table S7, documentation of progression before death by isotype within maintenance and treatment-era strata; Table S8, iFISH results by probe and isotype (positive, negative, not performed, missing); Table S9, sensitivity of the overall survival estimates to the censoring date of living patients. The STROBE checklist and the reproducibility package (analysis code) are supplied as separate files.

All results derive from the patient-level cohort of 196 patients described in Section 2.1 of the main text. Every value was generated by the analysis scripts in the reproducibility package (R/02_analysis.R, R/05_revision_analyses.R, R/06_revision2_analyses.R, and R/07_revision3_analyses.R) and copied from their output files.

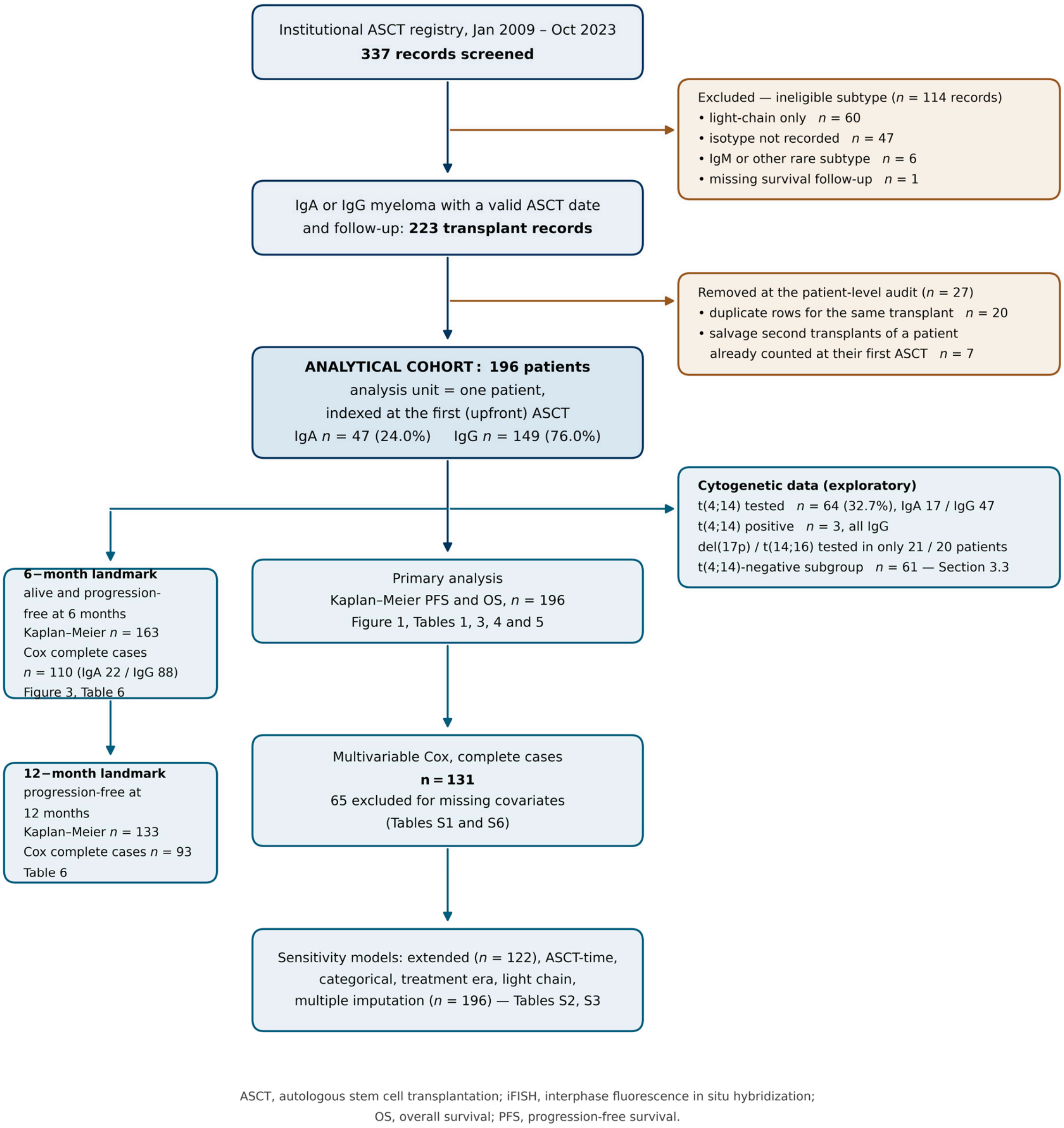

Figure S1. Patient selection flow chart. Flow of patients from the institutional transplant registry to the analytical cohort and to each analysis set. The analysis unit is one patient, indexed at the first (upfront) autologous stem cell transplantation. Records removed at the patient-level audit are shown separately from subtype-based exclusions. Cytogenetic denominators are given per probe (Section 2.4 of the main text).

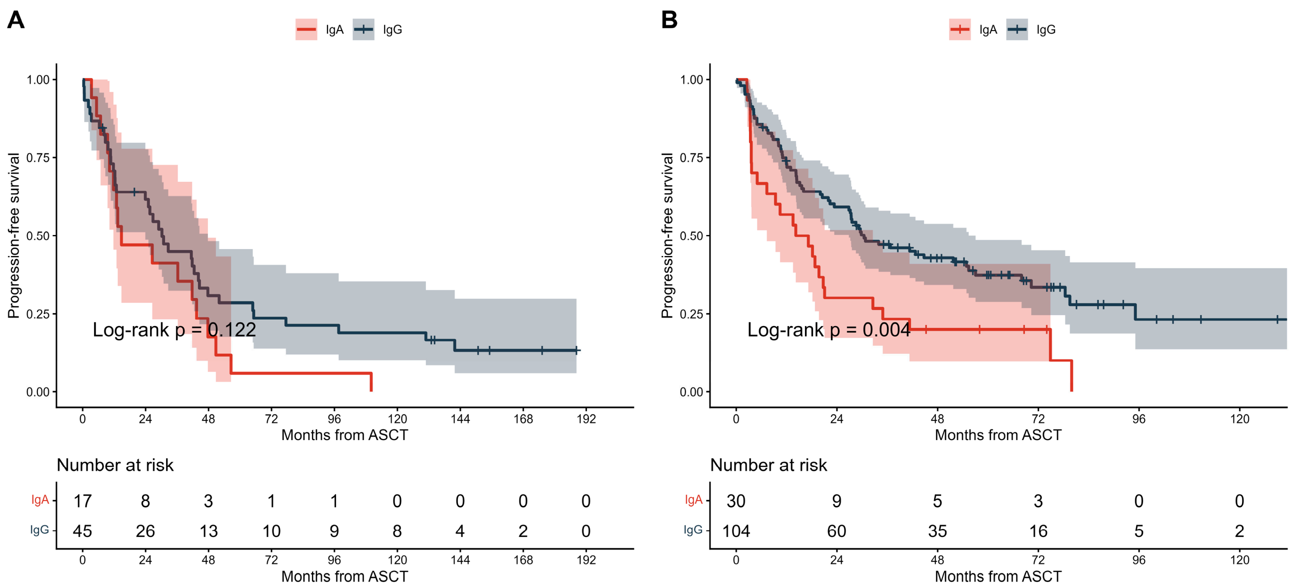

Figure S2. Era-stratified progression-free survival. Progression-free survival by isotype (A) before 2015 (IgA n = 17, IgG n = 45; median 14.8 versus 30.2 months, log-rank p = 0.122) and (B) from 2015 onwards (IgA n = 30, IgG n = 104; median 15.7 versus 30.5 months, p = 0.004), when lenalidomide maintenance was routine and its use was balanced between isotypes (IgA 76%, IgG 70%). Transplant accrual ended in October 2023, and the latest follow-up date in the registry was 29 April 2026, so a patient transplanted in January 2015 could be followed for up to 135 months; patients alive without progression are censored at the last recorded hospital visit.

Table S1. Missing covariate data and the complete-case restriction

The multivariable Cox models use the 131 of 196 patients with complete data for all base-model covariates.

Panel A. Missing values per covariate, whole cohort (n = 196)

Covariate	Missing, n	Missing, %	Used in the base model
IgA subtype	0	0.0	Yes (exposure)
ISS stage	15	7.7	Yes
Age at transplantation	1	0.5	Yes
Pre-ASCT response depth	50	25.5	Yes
Lenalidomide maintenance	1	0.5	Yes
Melphalan dose	2	1.0	Extended model only
Serum LDH	22	11.2	Extended model only
Light-chain type	0	0.0	Sensitivity model only

LDH, lactate dehydrogenase. Percentages are of the full 196-patient cohort.

Panel B. Pattern of missingness among the 65 excluded patients

Missing covariate pattern	Patients	% of exclusions
Pre-ASCT response only	48	73.8
ISS stage only	14	21.5
ISS stage and pre-ASCT response	1	1.5
Pre-ASCT response and maintenance status	1	1.5
Age at transplantation only	1	1.5
**Total excluded**	**65**	**100.0**

Missing data are largely due to the pre-transplant response assessment, which was not systematically documented in the early years of the study period.

Panel C. Complete-case status by immunoglobulin subtype

Group	Complete cases	Excluded	Excluded, %
IgA (n = 47)	30	17	36.2
IgG (n = 149)	101	48	32.2
**All (n = 196)**	**131**	**65**	**33.2**

Fisher's exact test p = 0.723. Complete-case status is not associated with the exposure, but excluded patients differ from included patients in the treatment period and induction regimen (Table S6); the multiple-imputation sensitivity analysis is in Table S3.

Table S2. Extended multivariable Cox models including serum LDH and melphalan dose

Complete cases for the extended covariate set: n = 122 (PFS 89 events; OS 64 events).

**Variable**	**PFS HR (95% CI)**	**p**	**OS HR (95% CI)**	**p**
IgA subtype (vs IgG)	1.77 (1.07–2.92)	0.026	1.84 (1.04–3.26)	0.036
ISS stage (per stage)	1.31 (0.96–1.78)	0.091	1.37 (0.94–2.01)	0.100
Age at ASCT (per year)	1.00 (0.97–1.04)	0.797	1.04 (1.01–1.08)	0.021
Pre-ASCT response (per category)	1.44 (1.04–1.99)	0.027	1.48 (1.03–2.13)	0.032
Lenalidomide maintenance †	2.38 (1.30–4.35)	0.005	1.19 (0.62–2.30)	0.598
Serum LDH (per doubling)	0.94 (0.57–1.56)	0.816	0.63 (0.33–1.21)	0.161
Melphalan 140 vs 200 mg/m²	0.53 (0.19–1.49)	0.229	0.51 (0.15–1.68)	0.265
C-index	0.621	—	0.630	—
Events	89	—	64	—

Serum LDH was log2-transformed, so its hazard ratio expresses the effect of a doubling. Neither LDH nor melphalan dose showed a statistically significant association with outcome, and their addition left the IgA estimate similar to that of the base model (PFS HR 1.87, OS HR 1.82; main text Table 2B). † The lenalidomide hazard ratio above 1 for PFS may reflect differential ascertainment of progression rather than harm; see Section 3.6 of the main text. Serum LDH was measured immediately before conditioning, after completion of induction, and therefore does not correspond to the diagnostic LDH used in the Revised International Staging System.

Table S3. Sensitivity models for the IgA effect and bootstrap validation of the concordance index

Panel A. Hazard ratio for IgA versus IgG under alternative model specifications

Model	n	IgA n	PFS events	PFS HR (95% CI)	p	OS events	OS HR (95% CI)	p
Base model (Table 2): IgA + ISS + age + response + maintenance	131	30	95	1.87 (1.17–3.01)	0.010	69	1.82 (1.07–3.09)	0.026
A1 IgA + ISS + age + response (no maintenance), same 131 patients	131	30	95	2.06 (1.29–3.30)	0.003	69	1.79 (1.07–3.00)	0.026
A2 IgA + ISS + age + response (no maintenance), all evaluable	131	30	95	2.06 (1.29–3.30)	0.003	69	1.79 (1.07–3.00)	0.026
B1 IgA + ISS + age + maintenance (no response depth)	179	41	136	1.76 (1.20–2.58)	0.004	102	2.10 (1.37–3.23)	0.001
B2 IgA + ISS + age (neither response nor maintenance)	180	42	137	1.90 (1.30–2.77)	0.001	103	2.05 (1.35–3.11)	0.001
B3 IgA + ISS + age + maintenance, restricted to the 131 complete cases	131	30	95	1.62 (1.03–2.56)	0.039	69	1.67 (0.99–2.80)	0.055
C1 ISS categorical (I ref), response categorical (CR ref)	131	30	95	1.78 (1.08–2.91)	0.022	69	1.84 (1.06–3.19)	0.029
C2 ISS categorical, response as CR vs less than CR	131	30	95	1.87 (1.14–3.05)	0.013	69	1.90 (1.11–3.28)	0.020
D1 Base + induction regimen (Vd ref / VCD / VRD-type)	131	30	95	1.84 (1.14–2.97)	0.012	69	1.84 (1.08–3.12)	0.024
D2 Base + year of ASCT (per year)	131	30	95	1.69 (1.03–2.76)	0.038	69	1.70 (0.99–2.91)	0.055
D3 Base + induction regimen + year of ASCT	131	30	95	1.68 (1.02–2.75)	0.041	69	1.72 (0.99–2.97)	0.052
D4 Within Vd-induced patients only, base model	59	14	50	1.15 (0.58–2.27)	0.696	39	1.44 (0.66–3.14)	0.363
D5 Within triplet-induced (VCD or VRD-type) patients, base model	72	16	45	2.72 (1.39–5.32)	0.003	30	1.65 (0.76–3.56)	0.202
E1 Base + lambda light chain	131	30	95	1.90 (1.18–3.06)	0.008	69	1.79 (1.05–3.06)	0.032
F1 Multiple imputation, base-model covariates (n = 196, m = 20)	196	47	147	1.82 (1.25–2.66)	0.002	110	2.12 (1.39–3.23)	0.001
F2 Multiple imputation, ASCT-time covariates (no maintenance)	196	47	147	1.98 (1.36–2.88)	<0.001	110	2.06 (1.36–3.11)	0.001

Base model covariates: IgA subtype, ISS stage (continuous), age, pre-ASCT response depth (ordinal), and lenalidomide maintenance. Models A use only information available at transplantation. Models B omit response depth; B3 refits B1 in the 131 complete cases to separate the effect of the covariate from that of the sample. Models C use categorical coding. Models D add induction regimen (bortezomib–dexamethasone as reference) and calendar year of transplantation (per year, centered at 2015); D4 and D5 are the base model within induction strata (IgA-by-triplet interaction: PFS p = 0.162, OS p = 0.863). Model E adds lambda light chain (lambda HR: PFS 0.87, 95% CI 0.55–1.37; OS 1.10, 0.65–1.84; IgA-by-light-chain interaction p = 0.887). Models F are multiple imputation by chained equations over all 196 patients (20 imputed datasets; predictive mean matching for age and LDH, polytomous regression for ISS, response and induction, logistic regression for maintenance and melphalan dose; the imputation model includes isotype, sex, treatment era, light-chain type and, for each outcome, the event indicator and the Nelson–Aalen cumulative hazard), combined by Rubin's rules.

Panel A2. Baseline-only model without maintenance (isotype, ISS stage, age, pre-ASCT response, induction regimen, and year of transplantation)

Analysis	n	IgA n	PFS events	PFS HR (95% CI)	p	OS events	OS HR (95% CI)	p
Baseline-only, complete cases	131	30	95	1.85 (1.13–3.02)	0.015	69	1.68 (0.98–2.88)	0.058
Baseline-only, multiple imputation (m = 20)	196	47	147	1.83 (1.22–2.73)	0.004	110	1.91 (1.24–2.94)	0.004

This is the main analysis of the manuscript (main text, Table 2A, which shows all coefficients); it was introduced during peer review and was not pre-specified. It combines the information available at transplantation and omits eventual receipt of maintenance, a post-baseline exposure. Induction regimen has bortezomib–dexamethasone as the reference; year of transplantation is per year, centered at 2015 (complete-case hazard ratios: 0.94 per year for PFS, 0.87–1.01; and 0.95 per year for OS, 0.87–1.04). The imputation model is that of model F, with year of transplantation in place of treatment era. Complete cases exclude 65 patients: 50 without a recorded pre-transplant response assessment and 15 with a recorded response but missing ISS stage (14) or age (1). With an age-by-log(time) term added (Table S5 footnote), the IgA hazard ratio was 1.74 (1.06–2.86) for PFS and 1.63 (0.95–2.79) for OS.

Panel B. Internal validation of the concordance index (200 bootstrap resamples, n = 131)

Model	Apparent C, without IgA	Apparent C, with IgA	Corrected C, without IgA	Corrected C, with IgA	Apparent difference (95% bootstrap percentile interval)	Optimism-corrected difference
PFS, base model	0.604	0.619	0.584	0.596	0.014 (-0.011 to 0.052)	0.012
OS, base model	0.603	0.622	0.574	0.591	0.019 (-0.008 to 0.068)	0.017
PFS, model without maintenance	0.598	0.609	0.581	0.593	0.011 (-0.011 to 0.063)	0.011
OS, model without maintenance	0.605	0.620	0.588	0.599	0.015 (-0.011 to 0.065)	0.012

Optimism was estimated as the mean over resamples of the difference between the model's concordance in the bootstrap sample, evaluated in that sample, and its concordance in the original data. The apparent difference compares the models with and without the isotype term in the original data; its interval is the 2.5th to 97.5th percentile of the same apparent difference across the bootstrap resamples, and it includes zero for all four comparisons. The optimism-corrected difference is the difference between the two corrected values and is reported only as a point estimate.

Panel C. E-values for unmeasured confounding

Endpoint (base model)	Adjusted HR	Approximate RR	E-value, point estimate	E-value, confidence limit
PFS	1.87 (1.17–3.01)	1.54	2.46	1.46
OS	1.82 (1.07–3.09)	1.51	2.39	1.28

Hazard ratios were converted to approximate risk ratios using $RR = (1 - 0.5^{\sqrt{HR}})/(1 - 0.5^{\sqrt{(1/HR)}})$, since both outcomes are common. The E-value is the minimum strength of association, on the risk ratio scale, that an unmeasured confounder would need with both isotype and outcome to explain the estimate fully (point estimate) or to move the confidence interval to include the null (confidence limit).

Table S4. Characterization of excluded registry records

Group	Records	Patients	Year of ASCT, median (IQR)	Before 2015, %	Age, median, y	Female, %	ISS recorded, n (% stage III)	Deaths	Follow-up, median, months
Light-chain only	60	58	2017 (2014–2020)	31	55.5	43.1	55 (56.4%)	31	52.3
Isotype not recorded	47	22	2021.5 (2017.5–2022.75)	13.6	53.8	36.4	7 (57.1%)	7	44.8
IgM / IgD / other rare	6	6	2011 (2011–2013.25)	83.3	61.5	33.3	4 (75%)	5	31

Patient counts apply the same de-duplication rule as the analytical cohort (first transplant per patient). Of the 47 records without a recorded isotype, 24 had no transplant date and are incomplete registry entries; the 22 patients with a transplant date were transplanted mainly from 2017 onwards, and had ISS stage recorded in only 7, consistent with incompletely entered records rather than a clinically distinct group. Light-chain-only patients were transplanted across the study period with outcomes similar to the analytical cohort (median follow-up 52.3 months; 31 deaths in 58 patients). The median year of transplantation for the 22 patients without a recorded isotype is 2021.5 because the number of patients is even. Follow-up is the median observed time from transplantation to death or last hospital contact.

Table S5. Covariate-specific Schoenfeld tests

Model	Term	χ^2	df	p
Base PFS	IgA	0.55	1	0.460
Base PFS	iss	1.00	1	0.317
Base PFS	age	6.24	1	0.012
Base PFS	resp	0.12	1	0.733
Base PFS	maint	11.73	1	0.001
Base PFS	GLOBAL	16.50	5	0.006
Base OS	IgA	1.49	1	0.222
Base OS	iss	0.12	1	0.731
Base OS	age	4.25	1	0.039
Base OS	resp	0.11	1	0.745
Base OS	maint	9.38	1	0.002
Base OS	GLOBAL	13.65	5	0.018
PFS, stratified on maintenance	IgA	0.00	1	0.986
PFS, stratified on maintenance	iss	0.97	1	0.325
PFS, stratified on maintenance	age	3.66	1	0.056
PFS, stratified on maintenance	resp	0.25	1	0.614
PFS, stratified on maintenance	GLOBAL	5.11	4	0.276
OS, stratified on maintenance	IgA	0.37	1	0.544
OS, stratified on maintenance	iss	0.02	1	0.893
OS, stratified on maintenance	age	2.53	1	0.112
OS, stratified on maintenance	resp	0.01	1	0.920
OS, stratified on maintenance	GLOBAL	3.09	4	0.543
ASCT-time PFS (no maintenance)	IgA	0.68	1	0.411
ASCT-time PFS (no maintenance)	iss	0.89	1	0.345
ASCT-time PFS (no maintenance)	age	7.71	1	0.005
ASCT-time PFS (no maintenance)	resp	0.23	1	0.634
ASCT-time PFS (no maintenance)	GLOBAL	8.74	4	0.068
ASCT-time OS (no maintenance)	IgA	1.44	1	0.231
ASCT-time OS (no maintenance)	iss	0.10	1	0.753
ASCT-time OS (no maintenance)	age	4.13	1	0.042
ASCT-time OS (no maintenance)	resp	0.12	1	0.734
ASCT-time OS (no maintenance)	GLOBAL	7.00	4	0.136

*Base models are those of the main text Table 2B (n = 131). The isotype term satisfies the assumption in every model. Stratification on maintenance status removes the maintenance violation and the global tests are no longer significant (PFS p = 0.276, OS p = 0.543), but it does not fully resolve the age finding: the age-specific test is p = 0.056 for PFS in the stratified model, and p = 0.005 (PFS) and 0.042 (OS) in the models without maintenance; a non-significant global test does not remove a covariate-specific violation. In the baseline-only model (main text Table 2A; Table S3, Panel A2), the age-specific tests were p = 0.010 (PFS) and 0.046 (OS). The age effect was therefore modeled as time-varying: coxph(Surv(t, event) ~ covariates + age + tt(age), tt = function(x, t, ...) x * log(t + 1)), with t in months since transplantation. The IgA hazard ratios were 1.78 (95% CI 1.11–2.85; p = 0.018) for PFS and 1.78 (1.05–3.02; p = 0.033) for OS in the base model, and 1.74 (1.06–2.86; p = 0.028) and 1.63 (0.95–2.79; p = 0.077) in the baseline-only model; the age-by-log(time) terms had p = 0.027, 0.039, 0.021 and 0.046, respectively.*

Table S6. Comparison of patients included in and excluded from the complete-case analysis

Characteristic	Included (n = 131)	Excluded (n = 65)	p
Patients, n	131	65	—
IgA isotype	30/131 (22.9%)	17/65 (26.2%)	0.723
Year of ASCT, median (IQR)	2017.0 (2015.0–2019.0)	2014.0 (2011.0–2017.0)	<0.001
ASCT before 2015	24/131 (18.3%)	38/65 (58.5%)	<0.001
Age, y	58.2 (52.9–62.9)	60.2 (53.1–64.5)	0.223
Female	57/131 (43.5%)	34/65 (52.3%)	0.288
ISS stage III (of recorded)	65/131 (49.6%)	33/50 (66.0%)	0.066
Induction Vd (of recorded)	59/131 (45.0%)	44/64 (68.8%)	<0.001
Lenalidomide maintenance (of recorded)	95/131 (72.5%)	37/64 (57.8%)	0.050
Serum LDH, U/L	187.0 (158.8–217.8)	201.0 (164.2–232.2)	0.191
Deaths	69/131 (52.7%)	41/65 (63.1%)	0.173
Progression or death	95/131 (72.5%)	52/65 (80.0%)	0.296
Median OS, months (KM)	78.4	55.1	0.310
Median PFS, months (KM)	27.4	27.6	0.323

Continuous variables: median (IQR), Wilcoxon rank-sum test; categorical variables: n/N (%), Fisher's exact test; medians of overall and progression-free survival by Kaplan–Meier, log-rank test. Excluded patients were transplanted earlier and more often received doublet induction, reflecting the period before structured response documentation, but did not differ materially in age, sex, stage, LDH, or outcome. The univariable IgA hazard ratio for PFS was 1.79 (95% CI, 1.14–2.81) in the included and 1.79 (95% CI, 0.98–3.28) in the excluded patients.

Table S7. Documentation of progression before death by isotype within maintenance and treatment-era strata

Among patients who died, the proportion in whom a progression had been documented before death. This is the ascertainment comparison of Section 3.6 and Table 6, broken down by treatment era (before 2015 versus 2015 onwards).

Era of ASCT	Lenalidomide maintenance	IgA, documented/deaths (%)	IgG, documented/deaths (%)	p (Fisher)
Before 2015	No	1/3 (33.3%)	4/15 (26.7%)	1.000
Before 2015	Yes	12/13 (92.3%)	12/14 (85.7%)	1.000
2015 onwards	No	4/5 (80.0%)	4/9 (44.4%)	0.301
2015 onwards	Yes	14/15 (93.3%)	31/35 (88.6%)	1.000

One IgA patient who died had no maintenance status recorded and is not represented. Documented progression is taken from the finalized first-event classification (main text Table 6). Documentation was lower without maintenance than with it in both eras. Within each era-by-maintenance cell, the isotype comparison was not statistically significant, but the cells without maintenance are small (3 to 15 deaths), and the proportions differ (for example, 4/5 IgA versus 4/9 IgG among non-recipients transplanted from 2015 onwards). These data suggest a maintenance-associated documentation difference but cannot exclude additional variation by isotype or era, and because they concern patients who died, they are not a direct measure of ascertainment among survivors.

Table S8. iFISH results by probe and isotype (positive, negative, not performed, missing)

Each probe was recorded in the registry as 1 (abnormality present), 2 (absent), 3 (probe not performed), or left blank. A probe counts as tested only when a result of 1 or 2 is recorded.

Probe	Isotype	Patients	Positive (1)	Negative (2)	Not performed (3)	Missing (blank)	Tested (1 or 2)
t(4;14)	IgA	47	0	17	4	26	**17**
t(4;14)	IgG	149	3	44	27	75	**47**
t(4;14)	All	196	3	61	31	101	**64**
t(14;16)	IgA	47	0	2	19	26	**2**
t(14;16)	IgG	149	0	18	56	75	**18**
t(14;16)	All	196	0	20	75	101	**20**
del(17p)	IgA	47	0	2	19	26	**2**
del(17p)	IgG	149	0	19	55	75	**19**
del(17p)	All	196	0	21	74	101	**21**

No other codes and no uninterpretable results were recorded. Neither code 3 nor a blank field was ever counted as a negative result: in the analysis dataset, the tested flag for each probe equals the presence of a code 1 or 2, a condition checked automatically (R/06_revision2_analyses.R, section F). The first build of the analysis dataset (August 2026) treated any non-blank value as a test result, so code 3 was read as tested and negative; this produced the figure of 95 patients with complete three-probe testing in the originally submitted manuscript. The coding was confirmed with the registry owner; code 3 was recoded as "not tested" in R/01_build_analysis_dataset.R, and all cytogenetic summaries in the current version (Table 1, Sections 2.4, 3.3, and 4.4, Figure S1) were regenerated using the corrected variables. All three probes were assessed in 20 patients (2 IgA, 18 IgG).

Table S9. Sensitivity of the overall survival estimates to the censoring date of living patients

No dated record of a cohort-wide vital-status query was retained. In the primary analysis, living patients are therefore censored for OS at the last date they were documented alive (the last recorded hospital visit or a later documented progression in one patient). The other rows censor every living patient at a common date instead: 29 April 2026, the latest date recorded in the registry, and 1 to 12 months later. These rows assume that every living patient survived to that date.

Living patients censored at	OS HR, base model (n = 131)	OS HR, baseline-only model (n = 131)	Reverse Kaplan–Meier follow-up, months	Median observed follow-up, months
Last documented date alive (primary)	1.82 (1.07–3.09); p = 0.026	1.68 (0.98–2.88); p = 0.058	86.2	47.7
29 April 2026 (survival assumed)	1.79 (1.07–3.02); p = 0.028	1.69 (0.99–2.89); p = 0.055	96.5	54.0
29 April 2026 + 1 month (hypothetical)	1.80 (1.07–3.02); p = 0.027	1.69 (0.99–2.89); p = 0.056	97.5	54.5
29 April 2026 + 3 months (hypothetical)	1.81 (1.08–3.05); p = 0.025	1.70 (0.99–2.91); p = 0.053	96.6	55.5
29 April 2026 + 6 months (hypothetical)	1.81 (1.08–3.05); p = 0.026	1.69 (0.99–2.89); p = 0.055	99.6	56.6
29 April 2026 + 12 months (hypothetical)	1.87 (1.11–3.15); p = 0.019	1.71 (1.00–2.94); p = 0.050	100.2	61.6

Held fixed in every row: all 110 deaths and their dates, the PFS data, and all covariates. Only the censored follow-up times of the 86 living patients change. Vital status was not re-ascertained at 29 April 2026 or at any later date, so the common-date rows describe the numerical sensitivity of the fitted models to the assumption of survival to that date; they are not additional observed follow-up and cannot establish when, or whether, vital status was verified. PFS is identical under every rule, because patients alive without documented progression are censored at the last recorded hospital visit. Three patients whose recorded progression date fell before transplantation or after death were not among the complete cases; excluding them from the full cohort gave univariable hazard ratios of 1.83 (1.28–2.63) for PFS and 2.05 (1.37–3.07) for OS (n = 193), and multiply imputed base-model estimates of 1.83 (1.24–2.70) and 2.19 (1.43–3.38).
